# *In silico* approaches to study the human asparagine synthetase: An insight of the interaction between the enzyme active sites and its substrates

**DOI:** 10.1371/journal.pone.0307448

**Published:** 2024-08-02

**Authors:** Anam Riaz, Afshan Kaleem, Roheena Abdullah, Mehwish Iqtedar, Daniel C. Hoessli, Mahwish Aftab

**Affiliations:** 1 Department of Biotechnology, Lahore College for Women University, Lahore, Pakistan; 2 Panjwani Center for Molecular Medicine and Drug Research, University of Karachi, Karachi, Pakistan; Holy Cross College, INDIA

## Abstract

Cancer is a leading concern and important cause of death worldwide. Cancer is a non-communicable illness defined as uncontrolled division of cells. It can develop into metastatic cancer when tumor cells migrate to other organs. In recent years evidence has emerged that the bioavailability of Asn play a crucial role in cancer metastasis. Asn is a non-essential amino acid formed from an ATP dependent catalyzed reaction by the enzyme asparagine synthetase (ASNS), where Asp and Gln are converted to Asn and Glu, respectively. The human ASNS enzyme consist of 561 amino acids, with a molecular weight of 64 KDa. ASNS governs the activation of transcriptional factors that regulate the process of metastasis. In this work the 3D model of ASNS in *E*. *coli* (AS-B) and the human ASNS docked with its different ligands have been used to study the 3D mechanism of the conversion of Asp and Gln to Asn and Glu, in human ASNS. The stability evaluation of the docked complexes was checked by molecular dynamic simulation through the bioinformatic tool Desmond. The binding residues and their interactions can be exploited for the development of inhibitors, as well as for finding new drug molecules against ASNS and prevention of metastatic cancer.

## Introduction

Cancer is a non-communicable disease, which is defined as the uncontrolled division of cells. In 2018 the World Health Organization (WHO) recorded 18.1 million new cases globally with 9.6 million deaths caused by cancer. In several high-income countries, the number of cancer cases has exceeded cardiovascular diseases accounting for twice as many deaths. Cancer development is a multi-step process, evolving from an *in situ* state and ultimately resulting in a malignant tumor [1]. It is most common in females, but has also been diagnosed in men [2, 3]. Breast cancer has the highest incidence rate of malignancy in women worldwide. The major clinical challenge faced by patients with breast cancer treated by conventional therapies is frequent relapse. Cancer metastasis depends on the primary tumor such as its physiology, anatomy and molecular features [4].

Asn is a nonessential amino acid, but it is required for protein synthesis in cellular growth and functions [5]. Asn regulates tumor progression at specific stages, and it has been shown that its bioavailability may regulate metastasis in breast cancer [6]. The enzyme asparagine synthetase (ASNS) generates the amino acid Asn from Asp. The gene encodes the ASNS enzyme, and is located on human chromosome 7. It consists of 13 exons, corresponding to the human ASNS protein of 561 amino acids. In the Uniprot database, 2 putative truncated isoforms of ASNS are found (isoform 2, residues (22–561) and isoform 3, residues (84–561). The enzyme is made of two domains: the *N*-terminal catalyzing the hydrolysis of Gln, and the *C*-terminal catalyzing the carboxylation of Asp [7].

A previous study has shown that ASNS performs a crucial role in cancer cell growth [5]. In breast carcinoma, inhibiting the ASNS expression results in low proliferation of cancer cells. In MDA-MB-231 breast cancer cell line (also known as triple negative cell), the hypoxia-circulating tumor cell, have the ability to induce the transcription factors ATF3 and ATF4 for ASN production [8]. In mice breast cancer models, high ASNS expression is positively correlated with the amount of circulating tumor cells and their invasive potential. The elevated epithelial-to-mesenchymal transition can partially explain how cell metastasis is promoted by ASNS, but more details still need to be investigated [9].

The 3D structure of the ASNS was first elucidated in *Escherichia coli (E*. *coli)* with a resolution of 2.0 Å. This structure contains two active sites. The first one, which is located in the *N*-terminal (also called the glutaminase domain or active site), catalyzes the conversion of Glu to Gln, and the second active site located in the *C*-terminal (also called the synthetase domain or active site) catalyzes the reaction, where Asp is converted to Asn. The binding of Mg^2+^ATP with Asp occurs in the synthetase domain of ASNS, which produces the β-aspartyl AMP intermediate. Furthermore Arg49, Asn74, Glu76 and Asp98 are found in the *N*-terminal active site form, which makes hydrogen bonds with Gln. Val272, Leu232, Gly347, Ser346, Asp238, Asp351, Asp279 and Asp384are located in the *C*-terminal active site, where ATP, Asp and other compounds potentially bind to ASNS [10]. Unlike the *N*-terminal active site, the *C*-terminal active site is not fully defined, and when the sequence of human ASNS was compared with that of *E*. *coli* ASNS, it was found that the synthetase domain (41.6% identity) in the *C*-terminal (residues 203–560) are more conserved than those in the *N*-terminal glutaminase domain (33.9% identity) (residues 1–202) [11].

In 2019, the human ASNS 3D model was determined [11]. It was done by using a compound called 6-diazo-5-oxo-L-norleucine (DON). It was suggested that different conserved residues (Arg48, Val52, Asn74, Gly75, Glu76, and Asp96) located in the *N*-terminal mediates substrate recognition and stabilize the hydrolysis reaction that produces ammonia. Furthermore, a cluster of negatively charged amino acids in the ASNS synthetase domain (Glu364, Asp367 and Glu368) were identified. They were suggested to play a key role in defining the binding selectivity of ASNS inhibitor 1 [11].

The use of bioinformatics tools significantly contributes to clinical and medical research. Computational biology has particularly shown to be useful in protein-based studies and for target identification. The significance of bioinformatics can be estimated from the largescale improvement of proteome analysis. Dry lab and wet lab experiments have proven to be innovative in biological research. Robotics and computational approaches provide valuable data to define target molecules in pharmacokinetics studies. In breast cancer studies, bioinformatics is used to predict protein 2D and 3D structures and also to evaluate the compatibility of drug molecules with the effected protein [12].

In this work the human ASNS enzyme’s active sites have been defined by using the known 3D structure of ASNS in *E*. *coli*. All substrates and other compounds involved in the conversion of Asp to Asn were used to dock with human ASNS 3D model. The binding residues have not yet been experimentally determined or predicted for human ASNS active sites, and this work can be further used for the development of therapeutic inhibitors.

## Materials and methods

### Retrieval of sequence data of ASNS protein

The FASTA sequence of ASNS protein was obtained from Uniprot (https://www.uniprot.org/) [13] with protein ID P08243 and P22106for Human and *E*. *coli*, respectively. These sequence data were used to determine the different regions of the protein and for docking studies.

### Sequence alignment for conservation analysis of ASNS in human and *E*. *coli*

The Clustal Omega (https://www.ebi.ac.uk/Tools/msa/clustalo) [14] tool was used to perform sequence alignment for determining the conservation status of amino acid between the human and *E*. *coli* sequences.

### Retrieval of 3D structure of ASNS protein

The 3D structure of human ASNS enzyme (PDB ID-6GQ3) was retrieved from RCSB PDB (https://www.rcsb.org/). This structure revealed the ASNS enzyme consists of two domains. The *N*-terminal domain (residue 1–208) and the second is *C*-terminal domain (209–561 residues).

### Active site identification of ASNS

The human (PDB ID6GQ3) and *E*. *coli* (PDB ID ICT9) ASNS 3D models were used to determine the binding sites in the active sites of ASNS. The LIGPLOT tool was used to represent the interaction between the protein and its ligands. LIGPLOT gives information about hydrogen bond interactions and hydrophobic contacts [15] (https://www.ebi.ac.uk/thornton-srv/software/LigPlus/download.html).

### Preparation of monomer target proteins

The human ASNS (target protein) structure was visualized and prepared utilizing the PyMOL visualization software (http://www.pymol.org/pymol) [16]. The gaps were removed from the 3D-structure and filled with missing residues found in the original FASTA sequence downloaded from Uniprot (https://www.uniprot.org/) [13]. This complete model was further used for molecular docking studies.

### Substrates of ASNS

The following ligands were used for docking with ASNS and to determine the binding site residues: ATP, Aspartate,β-Aspartyl, AMP and Glutamine. These ligands were used to dock with target protein as reference. This was used as a standard for finding potential residues of the enzyme active site.

### Preparation of target protein and ligand molecules

The human ASNS 3D model was prepared by removing all bound ligands and water molecules by using the discovery studio tool (https://discover.3ds.com/discovery-studio-visualizer-download) [17] and it was saved in PDB file format for further use.

2D sdf files of ATP, Asp and Gln were downloaded from PubChem database (https://pubchem.ncbi.nlm.nih.gov/) and their PDB files were created. β-Aspartyl AMP (the intermediate product of the enzyme reaction) was built by using the PyMOL builder system. All these 3D ligand molecules were saved in PDB file format.

### Preparation of PDBQT structures of target and ligands for docking

Energy refined PDBQT structures of target protein were prepared by addition of polar hydrogen molecules and kollmann charges by Autodock Vina [18, 19]. The ligand molecules were opened in Autodock Vina and torsion angles were adjusted. For the docking all the ligands files and target protein were converted to PDBQT file format.

### Molecular docking of the different substrates with the target protein

The Autodock Vina tool [18] was used to prepare all the required files to run docking command from command prompt. The grid box for target protein was developed by using the Autodock Vina grid box option with size of 40 Å for all three sides of box. The grid box dimension values for Glu (*N-*terminal binding) were 18.342, 16.339 and 27.450 for X, Y, Z, respectively. In case of ATP, AMP, L-Aspartate and β-aspartyl AMP (*C*-terminal bindings) X, Y, Z values were 16.868, 20.591 and -9.51. Different commands were used to accomplish the docking, output files and results models. Good binding energy (lowest affinity energy) models were selected and interactions were represented by using the PyMOL software. BioVia. The Discovery studio toolhttps://discover.3ds.com/discovery-studio-visualizer-download) [17] was used to analyze each docked complex for binding residues. The binding energy, distant length from ligands to residue atoms, amino acid residue and position of residues include the parameters, which were analyzed. Results were compared with the images from LIGPLOT and information about ASNS 3D models of both *E*. *coli* and human retrieved from the RCSB PDB database.

### Molecular dynamic simulation of docked complexes

For analyzing the simulations studies of the docked results, molecular dynamic (MD) simulations of protein-ligand docked complexes were performed. MD simulation was carried out for 100 nanoseconds by using Desmond, a package of Schrondinger LLC v3.6 module, version 2019 along with NPT ensemble class. All four docked complexes obtained from virtual screening results of Autodock vina were further used for the simulations; these docked complexes provide initial information about binding properties between ligand and protein, as it provides a fixed binding location for ligands with in protein binding pocket. Molecular docking studies are mostly performed to predict the interaction between the enzymes and its substrates in static conditions, while the molecular dynamic simulation studies predict these interactions in physiological conditions [20].

The protein preparation was achieved by minimization and optimization of protein ligand complexes. Protein preparation was done by the Wizard in Maestro tool. During the preparation all the distorted contacts, steric conflicts and deformed geometries were removed. Any extra molecules and water molecules were removed to make the structure clean as per simulation requirements. OPLS-2005 force field, TIP3P (Inter molecular interaction potential 3 points transferable) solvent model and orthorhombic box was used to build all the structures by using the system builder tool [21]. Simulation was performed at 300K temperature and 1atm pressure for 100 nanoseconds, 0.15M NaCl ions were added to maintain the ionic concentration at neutral charges. This ionic addition provides stability when opposite ions come in contact with models and to attain the physiological environment for the simulations [22]. The models were first relaxed and finally the molecular dynamic simulation was set for 100ns for all four docked complexes. MD simulated trajectories were analyzed after every 50ps. Desmond simulated MD trajectories were analyzed and stability of simulated complexes were determined by Cα root mean square deviation (RMSD), root men square fluctuations (RMSF), secondary structure element (SSE) and protein-ligand contact graphs.

## Results

### Comparison of human and *E*. *coli* FASTA sequences

The Uniprot portal was used to retrieve the FASTA sequence. The ASNS enzyme catalyzes three distinct chemical reactions: converting Gln to Glu, where ammonia is released, which occurs in the *N*-terminal domain. In the *C*-terminal active site Asp in reaction with ATP make the β-aspartyl-AMP intermediate, which reacts with the ammonia released from the *N*-terminal to produce Asn.

**Fig 1:**
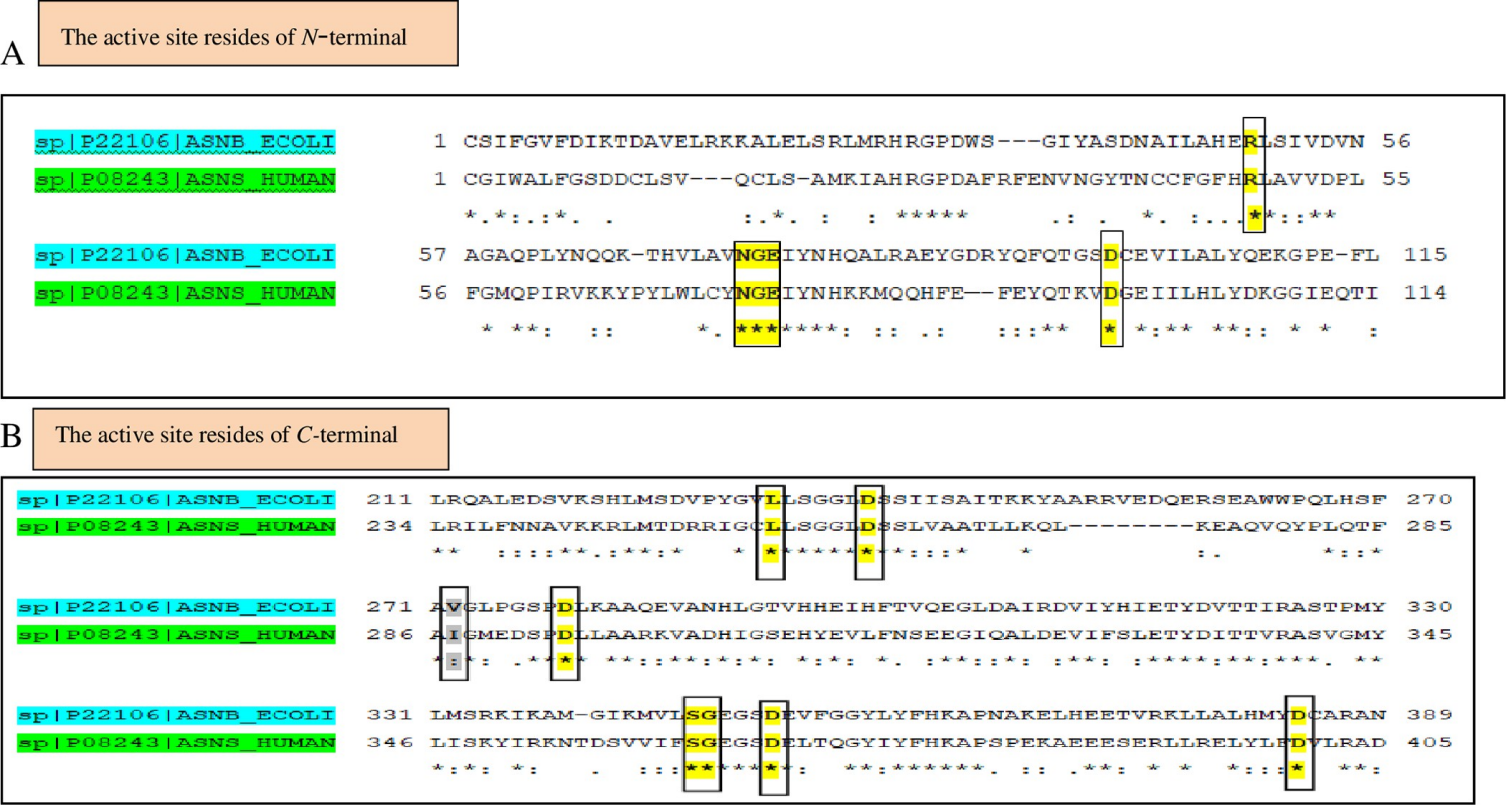
Sequence alignment between human and *E*. *coli*. (A) Showing that all the active site residues marked yellow of *N*-terminal are conserved. (B) Showing that all the active site residues marked yellow of *C*-terminal are conserved except Val of *E*. *coli* replaced with Ile of human: semi conserved marked gray.

The Clustal Omega tool http://www.ebi.ac.uk/Tools/msa/clustalo/ [14] was used to perform the alignment between two sequences of both organisms and the results suggest that both sequences have 37% percentage identity with11% gaps. The *N*- and *C*-terminal active site residues were found to be conserved in both organisms (Fig 1). All the active site residues located in the *N*- and *C*-terminal were found to be conserved except for Val at position 272 in *E*. *coli* has been replaced with Ile (position 287) in human ASNS as highlighted.

### 3D structure of target protein and its active site identification

To determine the specific residues that interact with the substrates in the active sites of ASNS the LIGPLOT software was used. The two models used for further comparison of the docking complexes were human ASNS docked with DON and *E*. *coli* AS-B docked with Gln and AMP [10]. The image of the human ASNS showed that in the *N*-terminal active site Arg48 (2.81Å), Asn74 (2.85Å), Asp96 (2.77Å) and Gly75 (3.01Å) are involved in the interaction with DON. The *E*. *coli* AS-B structure showed Gln binding with Arg49, Asn74, Asp98, Gly75 and Glu76 at *N*-terminal and AMP withSer234, Asp238, Ser346, Val272 and Glu352 at *C*-terminal site [11].

### Preparation of target protein and its ligands

**Fig 2:**
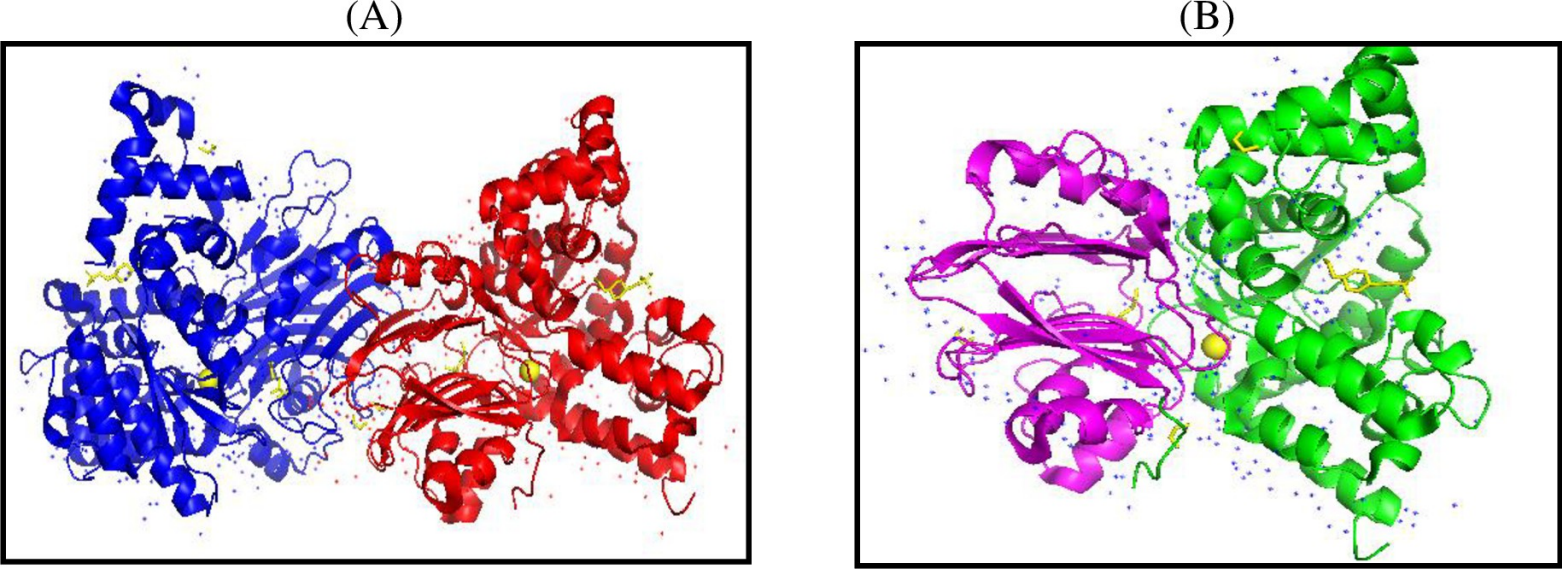
Preparation of monomer and gaps filling of missing residues in the PDB structure of target protein. (A) Dimer structure of the human ASNS protein with chain A (in blue) and B (in red). (B) Monomer structure of the ASNS protein with both domains (*N*-terminal in purple and *C*-terminal in green).

The PDB structure of the target protein (human ASNS) was retrieved from PDB with the ID 6GQ3. The head-to-head dimer structure of the enzyme was first converted to a monomer (Fig 2).

**Fig 3:**
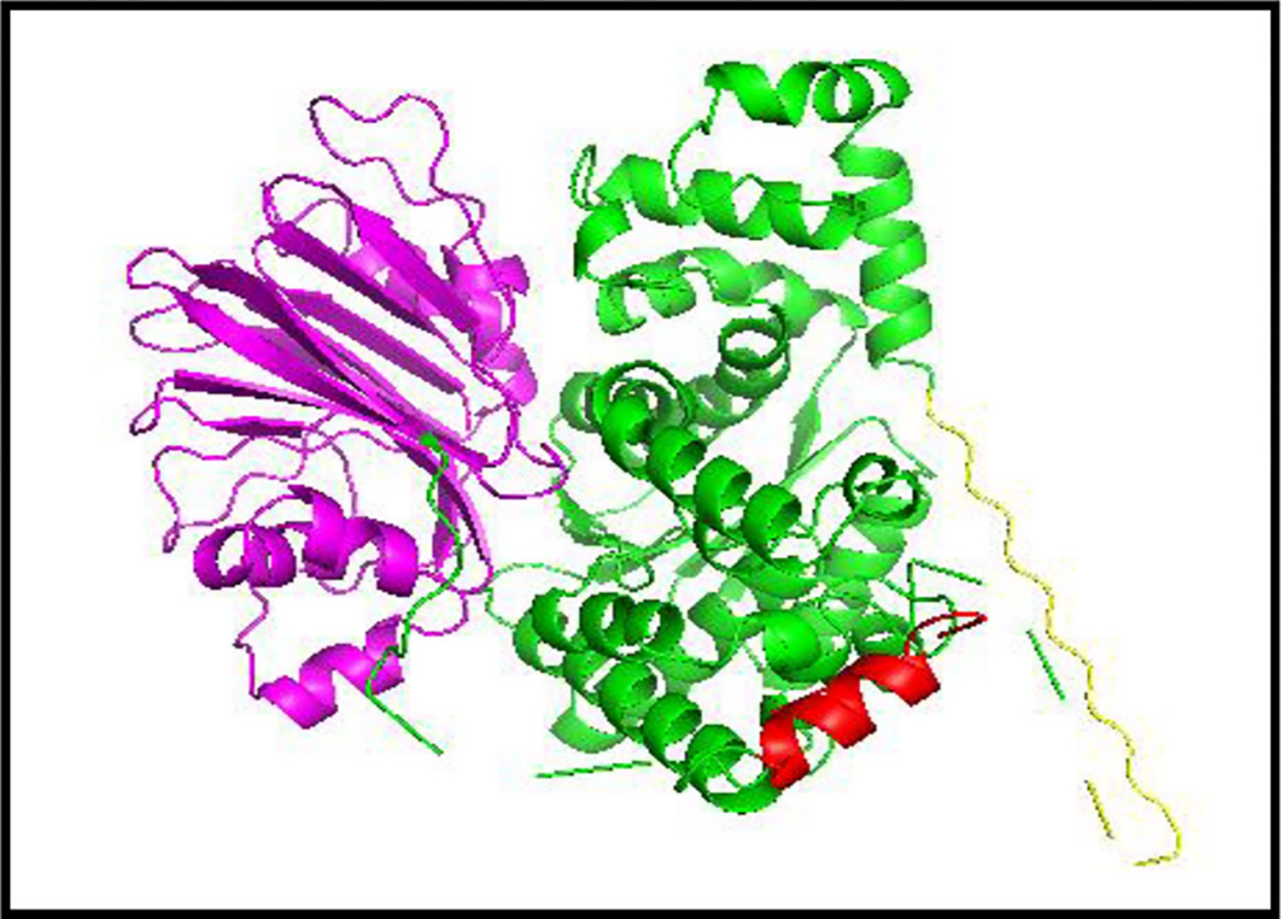
Water and ligand free PDB file of target protein: Showing both domains. (*N*-terminal in purple: *C*-terminal in green). yellow and red parts show the filled gaps in *C-*terminal.

The PyMOL builder software was used to fill the gaps with missing residues in the monomer target protein structure. The 12 missing residues Pro-Leu-His-Ala-Leu-Tyr-Asp-Ans-Val-Glu-Lys-Leu were added from position 210 to 221 and 13 missing residues Lys-Glu-Ala-Phe-Ser-Asp-Gly-Ile-Thr-Ser-Val-Lys-Asn were added from position 466 to 478 to make the *C*-terminal end complete (Fig 3).

**Fig 4:**
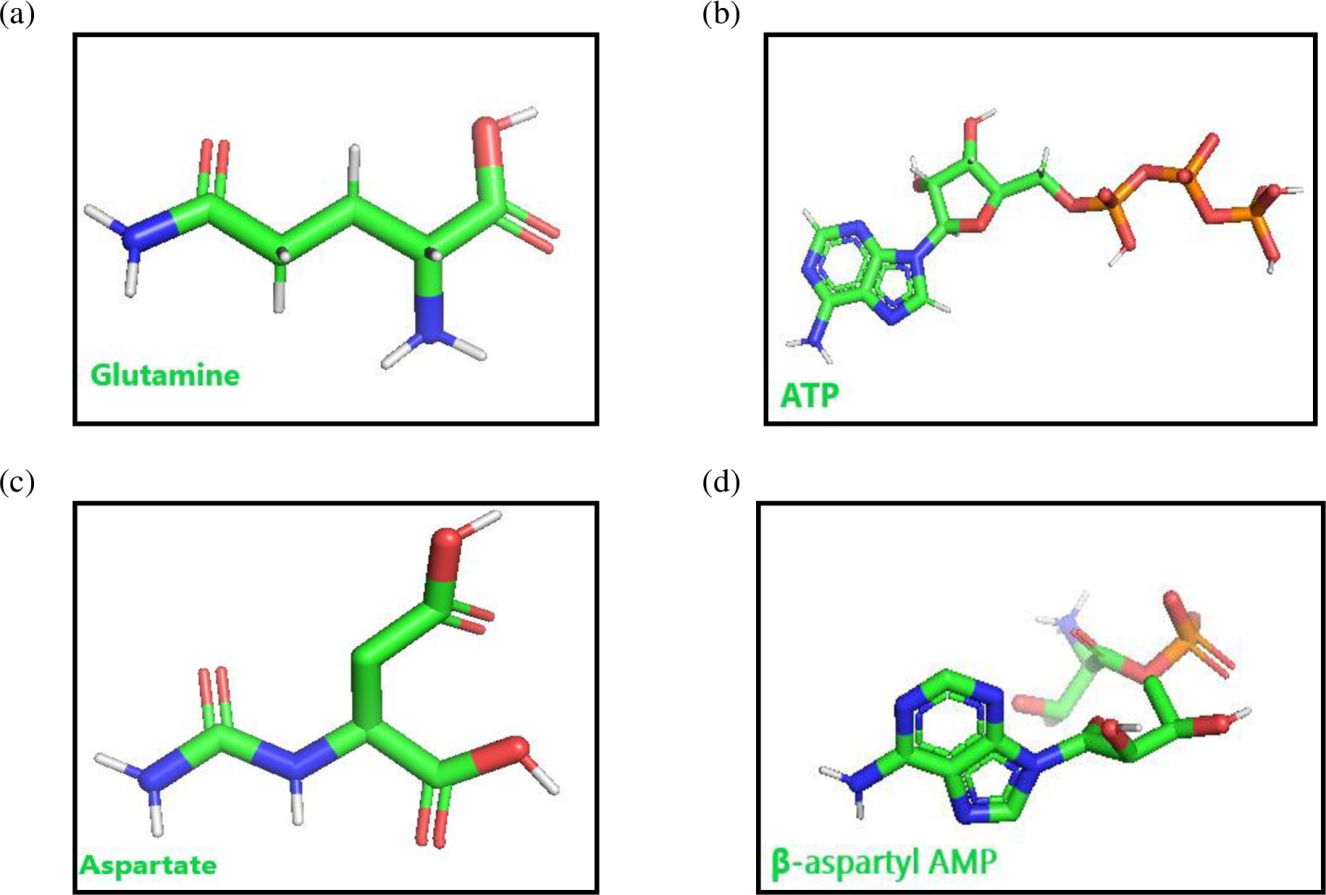
(a-d). 3D (Three dimensional) models of all four ligand molecules.

The Discovery studio tool (https://discover.3ds.com/discovery-studio-visualizer-download) [17] was used to prepare the target protein; all water molecules and bounded ligands were removed (Fig 3).The ligands ATP, Gln and Asp were downloaded from the PDB database, converted and saved in pdb format using the pymol software. The β-aspartyl AMP ligand was developed using and saved by PyMOL (Fig 4A–4D).

### Molecular docking of human ASNS target protein with its respective ligands

The energy minimized structures (PDBQT) of the target protein and all selected substrates were docked by using the Autodock Vina software. An output of 10 models was retrieved for each of the ligand molecules. Retrieval of log files with Vina’s output files, all docked complexes, their binding energy, RMSD (root mean square deviation) values and interaction were obtained (S1-S4 Tables in S2 File). Best models were selected on the basis of their binding energy: The lower binding energy indicates a higher binding affinity and stability of docking results. Bond length between ligand molecules e.g. ATP and residues of target protein were also used to for this study. RMSD gives information about the distance between two molecules, the models with smaller RMSD were considered as best model (Table 1). All these results were compared with the information derived from the human and *E*.*coli* ASNS structures.

**Table 1:** Binding energy and RMSD of selected docking complexes.

Docking Complex	Model No	Binding Energy (kcal/mol)	RMSD	
			Upper bound	Lower bound
ASNS with Gln	01	-5.7	0.000	0.000
ASNS with ATP	01	-9.1	0.000	0.000
ASNS with Asp	01	-5.8	2.236	4.1021
ASNS with β-Aspartyl AMP	01	-8.1	0.000	0.000

### Molecular docking of Gln with human ASNS target protein

**Fig 5:**
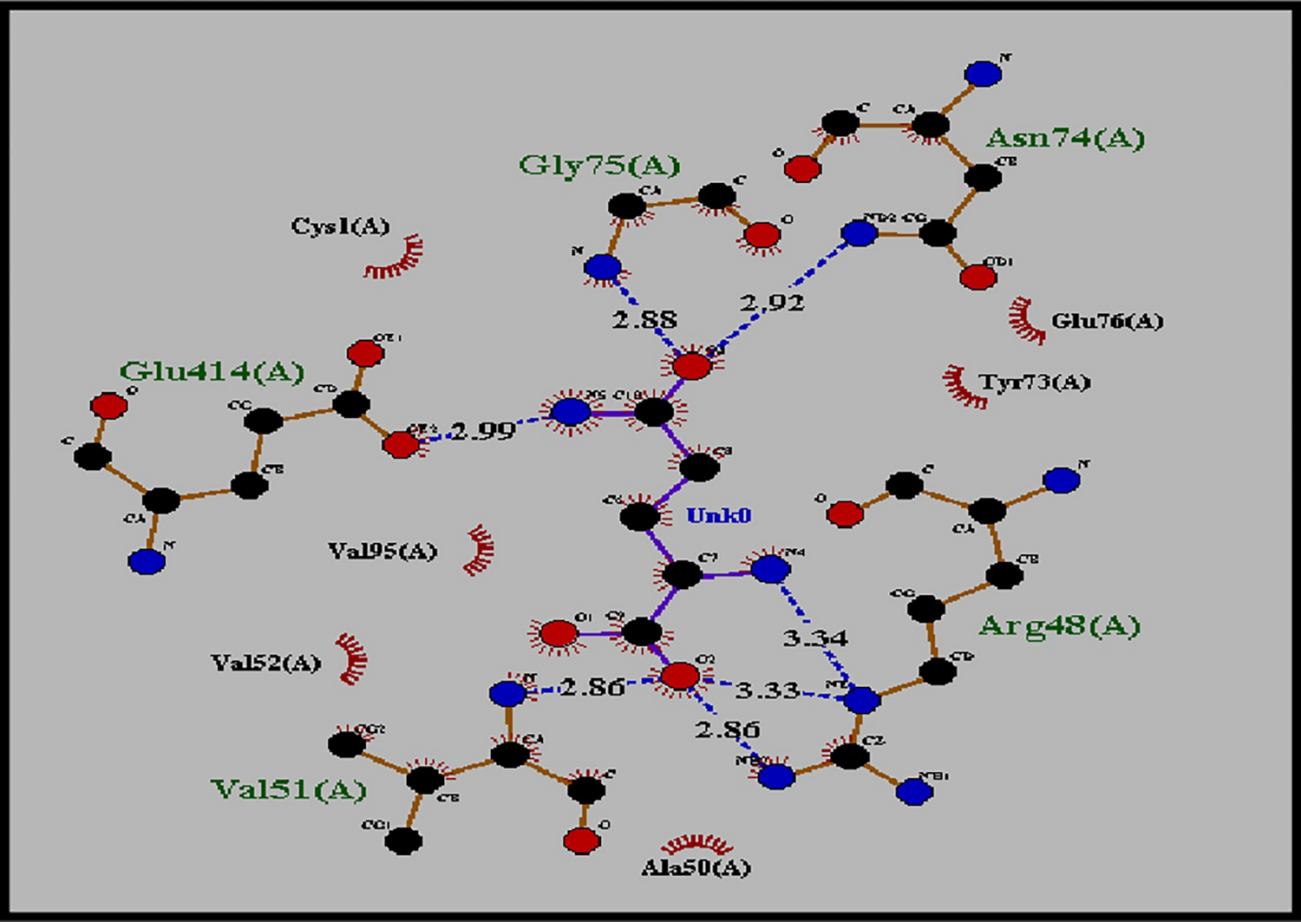
Ligplot showing hydrogen bonding and hydrophobic interaction between the Gln ligand and ASNS. Gln binds via hydrogen bonds with ASNSat Arg48 with 2.86Å, Asn74 with 2.92Å, Gly75 with 2.88Å and Glu414 with 2.99Å, and via hydrophobic interactions with Val52, Ala50, Val95, Cys1, Glu76 and Tyr73.

The docked complex of Gln with ASNS target protein were obtained from docking results used for analysis based on their minimum binding energy (S1 Table in S2 File and S1 Fig in S1 File). The binding energy of the selected model was -5.7 kcal/mol, and the RMSD values from lower bound and upper bound were 0.000 (Table 1). The LigPlot image shows that Gln binds at the glutaminase binding site in the *N-*terminal domain (Fig 5). The hydrogen bond interactions are represented with blue dashed line including the distance length in angstrom, while the hydrophobic interactions are shown by an arc. The hydrogen bond interacting residues are Arg48, Asn74, Gly75, Val51 and Glu414 and hydrophobic interacting amino acids included Ala50 Val95, Tyr73, Cys1, Glu76 andVal52 (Fig 5). This is in agreement with the *E*. *coli* AS-B structure, where it has been shown that the amino acid Gln binds with Arg49, Asn74, Asp98, Gly75 and Glu76 at *N*-terminal [10]. The amino acid Glu414 also binds with the ligand Gln, as this residue provides a linkage following the amidotransferase reaction [11].

### Molecular docking of ATP with human ASNS (*C-*terminal) target protein

**Fig 6:**
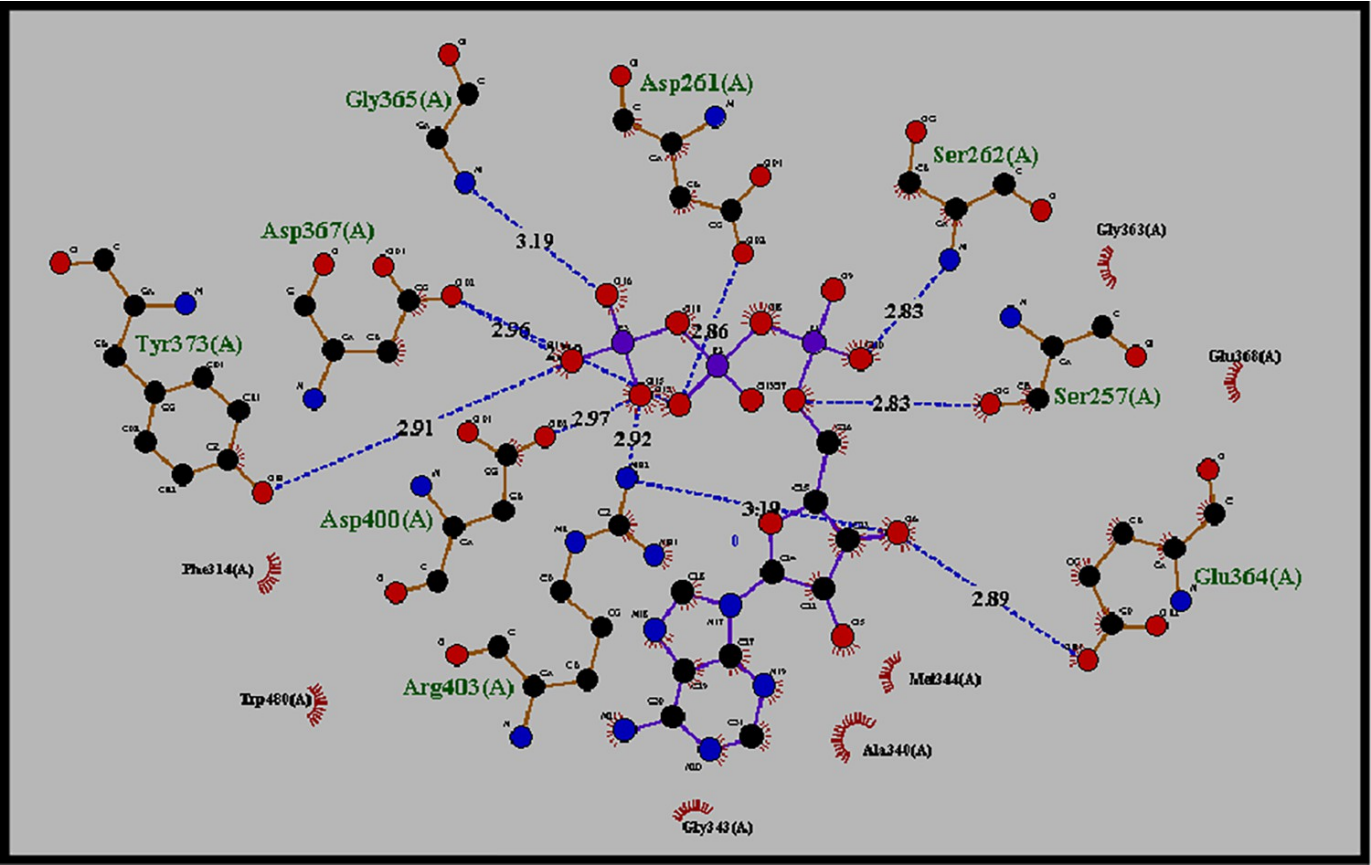
Ligplot showing hydrogen bonding and hydrophobic interaction between the ATP and ASNS protein. ATP binds with ASNS at Ser257 with 2.83Å, Glu364 with 2.89Å, Ser262 with 2.83Å, Asp261 with 2.86Å, Asp367 with 2.70Å and Asp400 with 2.97Å and via hydrophobic interactions with Glu 368, Phe314, Trp480, Gly343, Ala340 and Met344.

The docked complex of ASNS target protein and ATP with minimum binding energy was selected for analysis (S2 Table in S2 File, S2 Fig in S1 File). The minimum binding energy of selected models was -9.1 kcal/mol, and RMSD values from lower bound and upper bound were 0.000 (Table 1). ATP binds at the synthetase/ATP binding site in the *C-*terminal domain of target protein, where hydrogen bonds and hydrophobic interactions were observed on a Ligplot image (Fig 6). The hydrogen bond interacting residues were Ser257, Asp261, Ser262 Glu364, Gly365, Asp367, Tyr373, Asp400, and Arg403 and hydrophobic interacting amino acids included Glu368, Gly363, Phe314, Trp480, Gly343, Ala340 and Met344. The atoms of ligand molecule along with distant length in angstrom such as ATP bindwith Ser257 (2.83 Å), Ser262 (2.83Å) and Glu364 (2.8Å).

### Molecular docking of Asp with human ASNS (*C-*terminal) target protein

**Fig 7:**
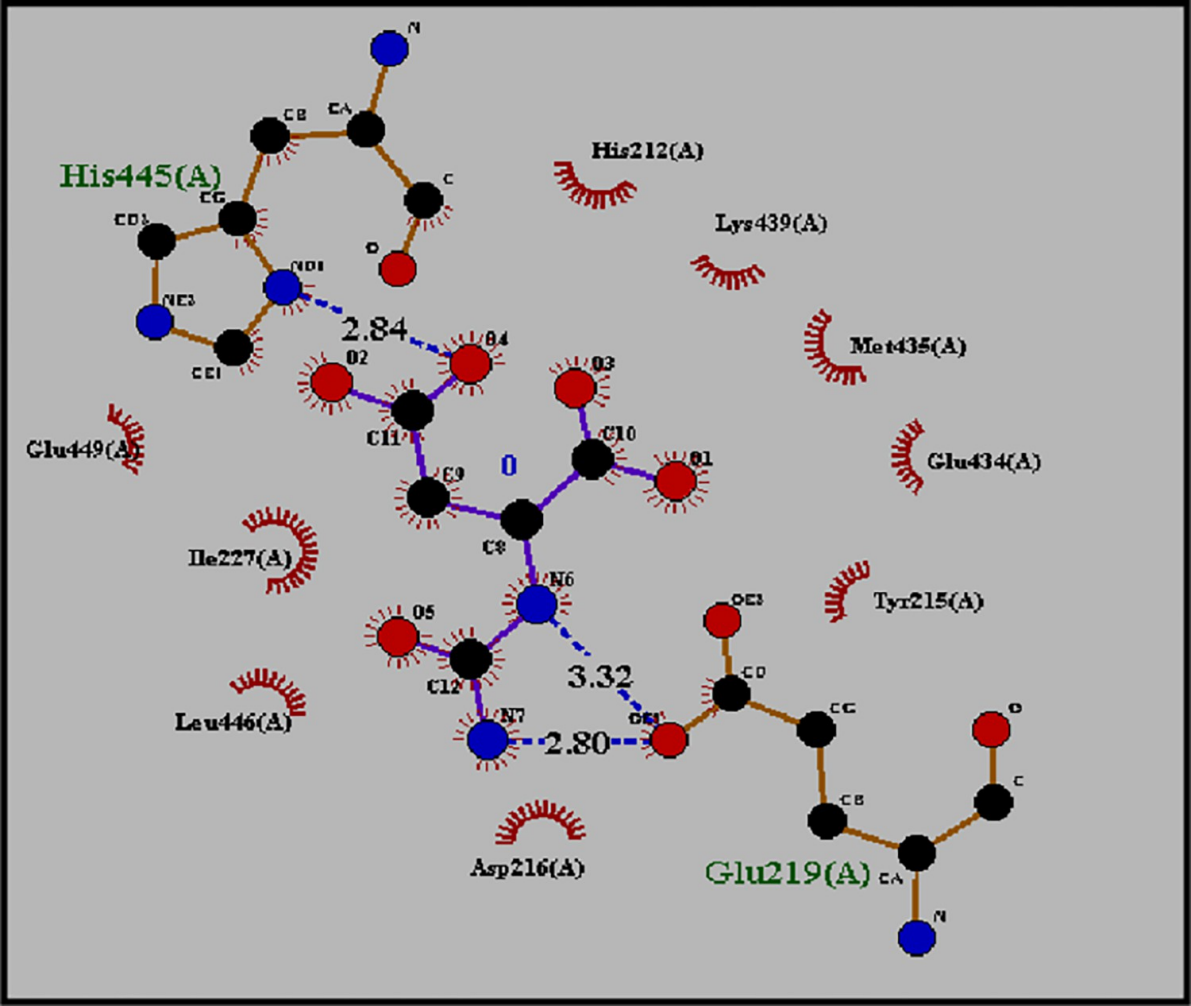
Ligplot showing hydrogen bonding and hydrophobic interaction between the Asp ligand and ASNS protein. Asp binds with ASNS at His445 with 2.84Å, and Glu219 with 2.80Å and via hydrophobic interactions with Leu446, Ile227, His212, Asp216, Lys439 and Met435.

The docked complex of Asp with ASNS target protein binding energy was -5.8 kcal/mol and RMSD values from lower bound and upper bound were 2.236 and 4.102, respectively (Table 1). This model was selected on the basis of the binding between Asp and ASNS (S3 Table in S2 File and S3 Fig in S1 File). The first model, which has lowest binding energy, and binding of Asp with Met occurs with the alpha carbon carbonyl group, which interrupt the peptide bonding in ASNS. The hydrogen bond interacting residues are His4459and Glu219while His212, Asp216, Lys439, Met435, Glu434, Glu449, Ile227 and Leu446 show some hydrophobic attractions (Fig 7). The LigPlot image of Asp and ASNS docked complex in which blue dashed lines represents the H bonding between residues of target protein and atoms of ligand molecules along with distant length in angstrom. such as in complex where Aspbinds at His445 with 2.84Å.

### Molecular docking of β-Aspartyl AMP with human ASNS (*C-*terminal) target protein

**Fig 8:**
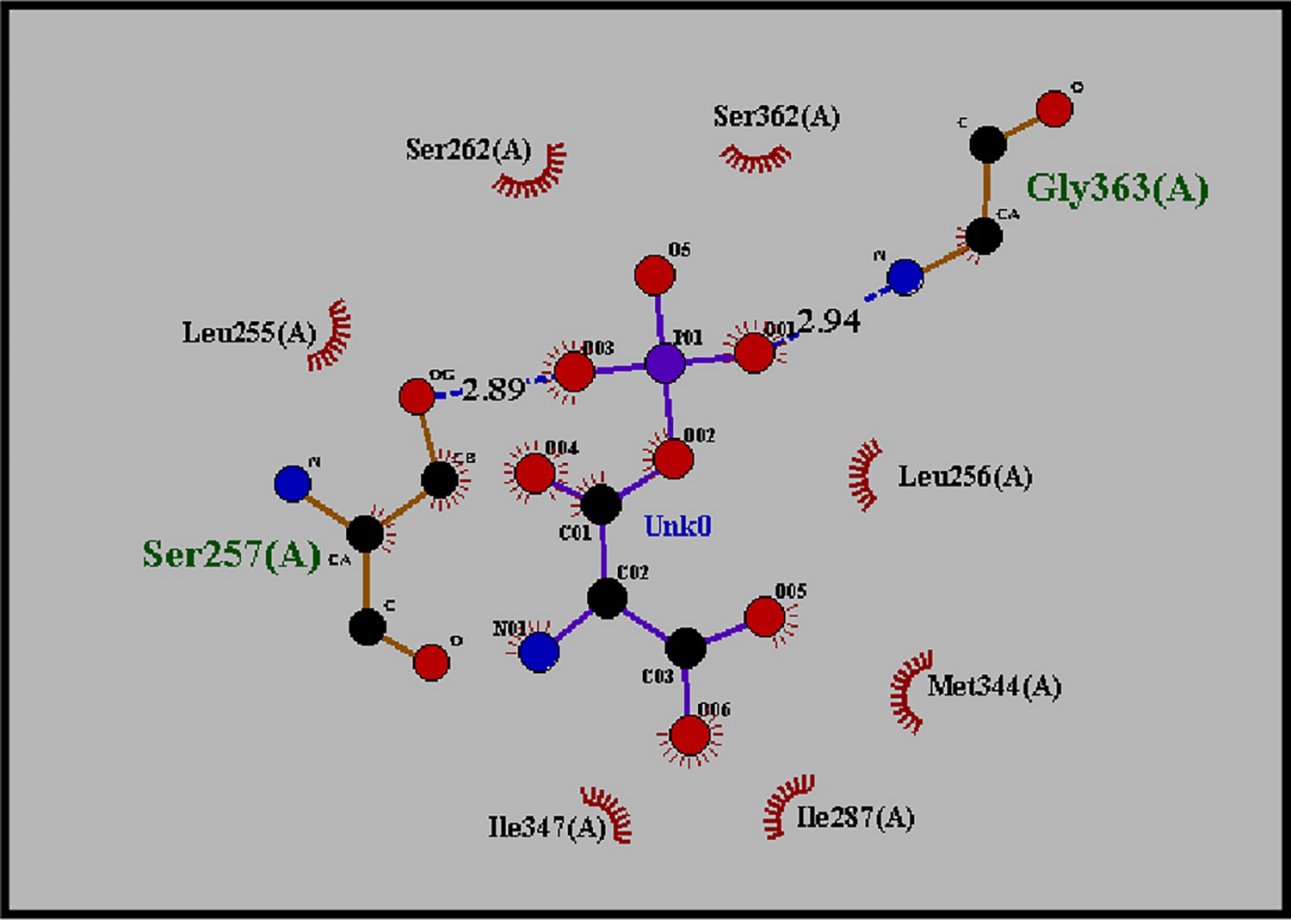
Ligplot showing hydrogen bonding and hydrophobic interaction between the β-Aspartyl AMP ligand and ASNS protein. β-Aspartyl AMP binds with ASNS via hydrogen bond at Gly363 with 2.94Å and Ser257 with 2.89Å and via hydrophobic interactions with Ile347, Ile287, Met344, Ser262 and Ser362.

The docked complex of β-Aspartyl AMP and ASNS target protein and with minimum binding energy was selected from output models obtained from docking results for analysis. The binding energy of selected models was -8.1 kcal/mol which is minimum above all and RMSD values of model 01 from lower bound and upper bound were 0.000 which tells the distance from best model (S4 Table in S2 File and S4 Fig in S1 File). β-Aspartyl AMP is an intermediate of the overall reaction and binds at synthetase/ATP binding site in *C-*terminal domain of target protein. The hydrogen-bond interacting residues are Ser257, Gly363 and hydrophobic interacting residues are Ser262, Ser362, Leu256, Met344, Ile287, Ile347 and Leu255 (Fig 8). The hydrogen bond interactions are represented by blue dashed line including the distance length in angstrom while the hydrophobic interaction represented by an arc in the Ligplot image such as β-Aspartyl AMP binding at Ser257 with 2.89Å.

### Stability evaluation of docked complexes

**Fig 9:**
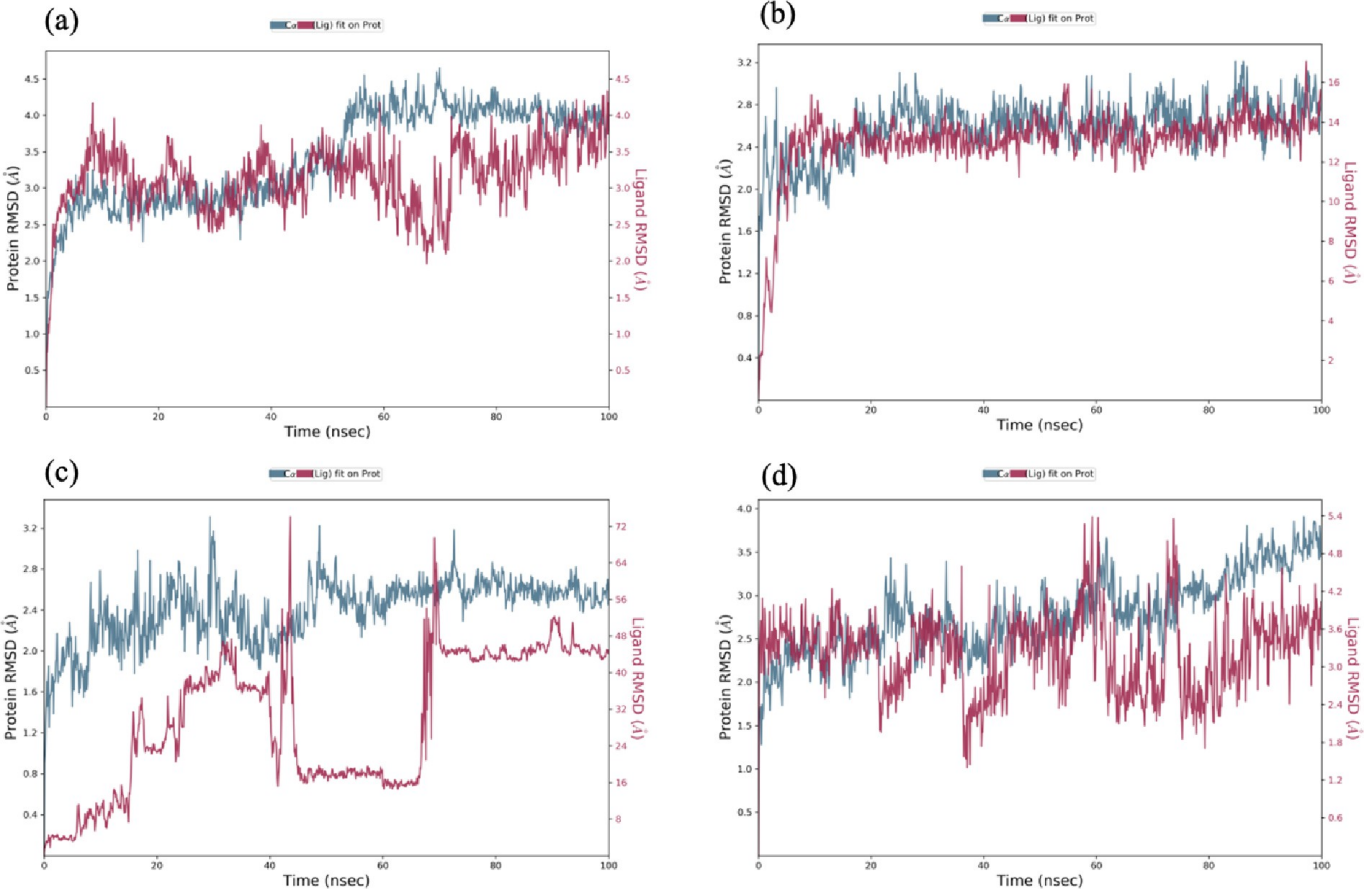
RMSD evaluations of docked complexes between the C-alpha atoms of proteins and ligands in 100ns simulation. The pink shows the RMSD of the ligands compound and blue color shows the RMSD of the protein (a) shows RMSD plot between protein and ATP, (b) shows the RMSD plot of beta aspartyl AMP (c) shows the plot between Asp and protein and the (d) shows the RMSD evaluation between protein and Gln ligand.

MD simulated trajectories were analyzed and RMSD, RMSF, SSE and protein ligand contacts were calculated for evaluation of complex stability. Docked complexes were simulated using Desmond molecular dynamic simulation for 100ns. Fig 9 shows the graphs for the estimation of RMSD value on y-axis with respect to time in nanoseconds on X-axis for Cα particles in protein ligand docked complexes. Graphs were plotted for all four complexes (a:ASNS-ATP), (b:ASNS-beta aspartyl AMP), (c:ASNS- Asp) and (d:ASNS-Gln) respectively, where the blue line (left side of y axis) represents the protein, while the pink lines (right, y-axis)represent RMSD evaluation of the ligand.

RMSD plots shows that all the docked complexes are stabilized at the start of the simulations. The simulation plot of the protein-ATP complex reaches at stability at 10ns, followed by small variation after 70ns and become equilibrated at 80ns and remain stable till the end of the simulation. Similarly, Fig 9B and 9D also show that the complexes are stable during the simulation, whereas Fig 9C the docked complex between Asp and protein show that the ligand stability is delayed (at 40ns) becomes stable at 80ns and remain stable till end of the simulation. The average RMSD values were found to be 3.2Å, 2.5Å, 2.7Å and 2.4Å, respectively. Ligand fit to protein RMSD values remains within range after being stable, which indicates that all these ligands remain stable at the binding site of the protein during the simulation.

**Fig 10:**
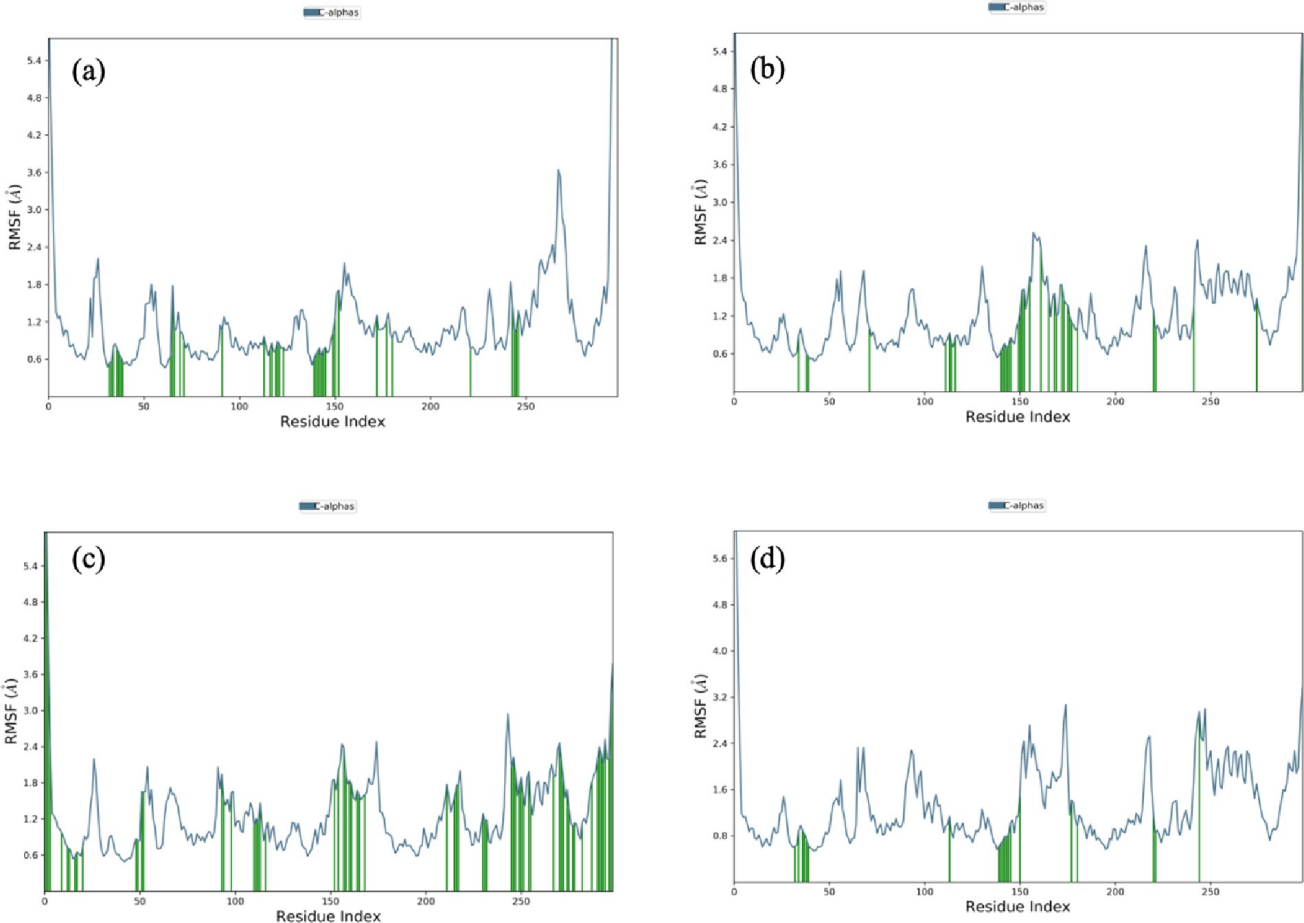
Residue wise Root Mean Square Fluctuations (RMSF) graphs of the docked complexes. (a) RMSF plot of docked complex of ATP with protein. (b) RMSF plot of docked complex of beta aspartyl amp with protein. (c) RMSF plot of docked complex of Asp with protein. (d) RMSF plot of docked complex of Glu with protein.

RMSF plots were used to determine the residues fluctuations of the protein bound to its respective ligands. Peaks of RMSF plot indicate the fluctuated area of the protein during the simulation. The *N*- and *C*-terminus regions are relaxed, which gives more fluctuations, while the protein configuration consisting of alpha helices and beta sheets are usually more rigid and show fewer fluctuations during the simulation. RMSF graph were plotted for all four docked complexes (Fig 10).

**Fig 11:**
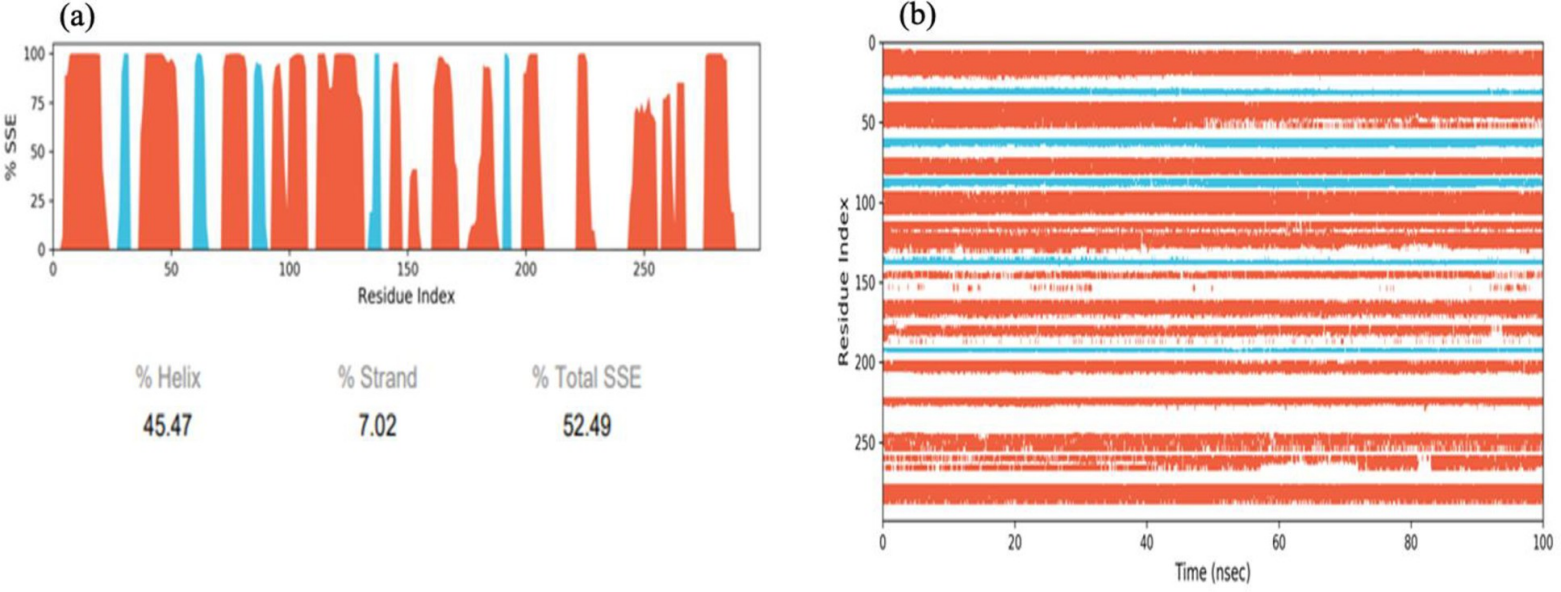
Distribution of SSE of the protein over the time of the simulation. (a) Shows the overall distribution of SSEs. (b) Shows each residue with SSE assignment. The alpha helices are represented by the red columns and the beta strand are colored blue.

RMSF plot of the docked complexes show some higher peaks (>3.0 Å), which indicated those residues which present in loops in the *N*-and *C*-terminus regions, whereas the lower peaks (<3.0 Å) suggest the presence of rigid secondary structures such as alpha helix and beta sheets. Furthermore, a low RMSF value indicates that over all protein-ligand complexes are stable and show consistency of ligands towards the protein residues. The green bars represent those residues which are in contact with the ligand and a low RMSF values indicate their strong binding ability (Fig 10A–10D). Secondary Structure Element (SSE) includes alpha-helices and beta-strands determined during the simulation (Fig 11). The protein SSE histogram illustrates that 52% residues account for alpha-helices and beta-strands from the total protein structure (Fig 11).

**Fig 12:**
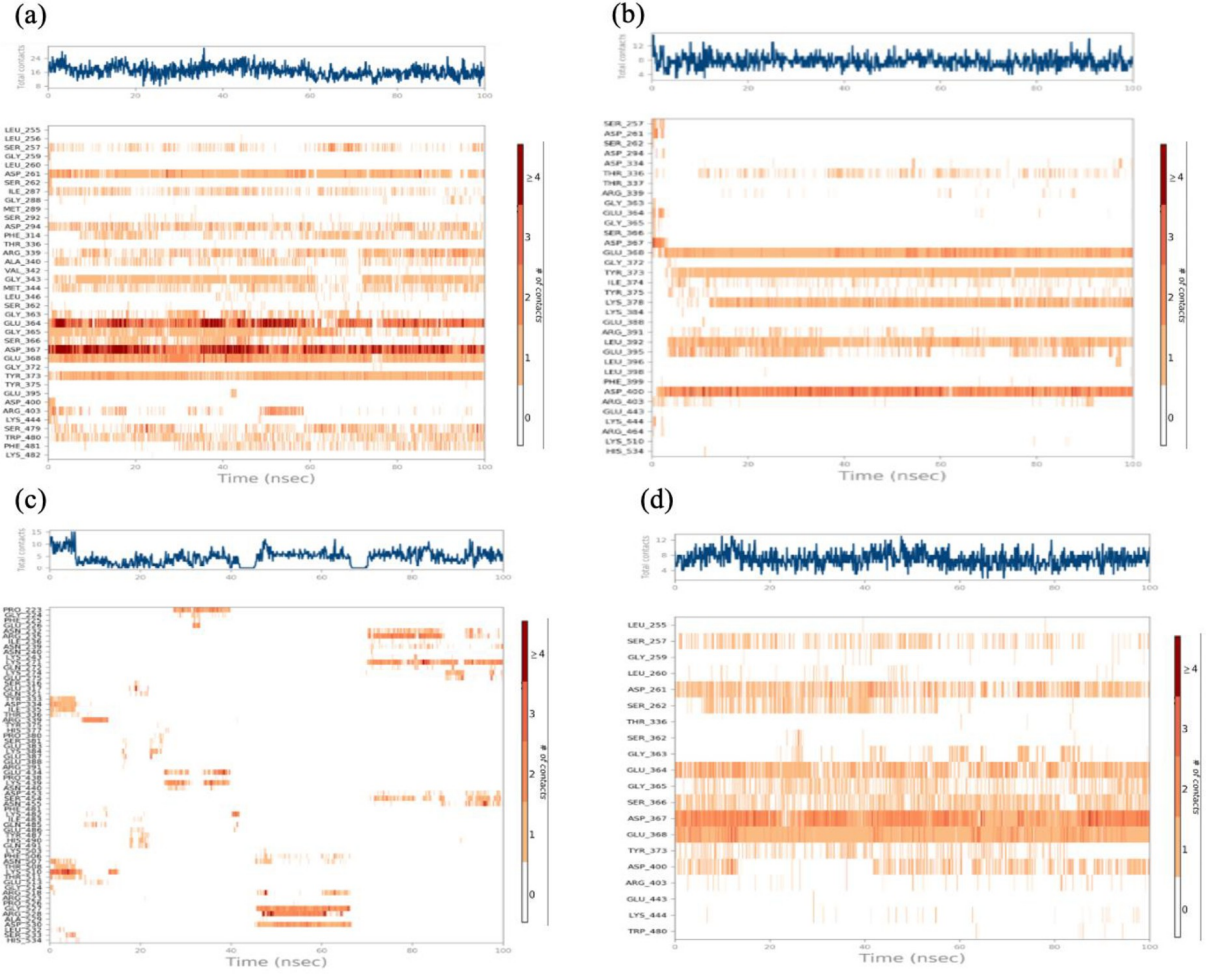
Protein-ligand contact and heat map timeline throughout simulation. (a) Shows protein ligand contacts between the ATP and target protein. (b) Showing the interactions of beta aspartyl amp with target protein. (c) Shows the contacts between Asp and protein. (d) Depicts the protein ligand contacts of Glu with target protein.

The protein ligand contacts were also monitored during molecular dynamic simulation. Protein ligand interactions are of different types such as hydrogen bonds, ionic bonding, water bridges and hydrophobic interactions. Each of the residues that bound to the ligands attained specific contact type, which can be determined during the simulation. The top graphs (blue line) of protein-ligand plot illustrates the maximum number of contacts of all four ligands with the target protein during 100ns simulation time over the course of MD trajectories (Fig 12A–12D). The bottom panel (orange bars) of the graphs show the heat map timeline that defines the protein-ligand contacts showing the residues that make interaction towards the ligand in MD trajectory for each frame in the given simulation. Residues (dark orange) show that they have more than one type of independent contact or interaction with the ligand (Fig 12A–12D). The highest number of contacts was observed, when ATP was docked with ASNS (24 contacts). Specifically, the residues Asp261, Gly363, Glu364, Asp367 and Thr373 located in the *C*-terminal binding pocket of ASNS show stronger interactions due to their darker color.

## Discussion

In nature an enzyme usually folds into a specific 3D structure and performs a unique function required at the given time [23]. To determine the specific structure of a protein or enzyme is essential to depict the function and to understand the biological processes and pathology of cancer. The dry lab techniques are now unavoidable to investigate the 3D structure of protein for classification, prediction of function for uncharacterized proteins, interaction with other macromolecules, structure-based drug development, interactions with small ligands like metal ions, nucleotides, substrates, cofactors and inhibitors and predicting active site for targeted therapy [24]. Currently, more than 200,000 structures are submitted in the PDB, which include proteins, nucleic acids, carbohydrates, and lipids and this number is constantly growing [25]. In this work the structural organization of the catalytic and substrate binding site of human ASNS has been determined. This will help in understanding the interactions between enzyme and its substrates and their structural properties.

Enzyme active sites are the regions usually located on the surface, where substrates bind and catalyze a specific reaction. Amino acids of the active site are known to be highly conserved, which is useful in determination of their functional importance. Numerous methods based on the structure of these active sites have been proposed such as the GASS WEB database, which is used to depict or find active sites in enzymes [26].

Asn is a non-essential amino acid widely required for cellular growth and functions. Cells receive Asn physiologically through two major ways: Circulating ASNS or plasmatic Asn. The cytoplasmic enzyme ASNS converts Asp to Asn and Gln to Glu in the presence of ATP. ASNS is abundantly found in many organs of mammals, but its basal expression varies being highest inthe pancreas, brain, thyroid and testis, while lowest in the liver [27]. In human the activity of this enzyme is greatly influenced by different cellular stresses, and through transcriptional regulation, it becomes target of two different signaling pathways, one which ensure cell survival (Amino Acid Response) [28] and the other, which is caused by increased endoplasmic reticulum stress (the Unfolded Protein Response) [29]. The *ASNS* gene can also be regulated by other factors like the transcription factor p53, which has been shown to negatively control the *ASNS* expression in lung cancer [30].

The importance of ASNS in cancer metastasis is evident. In human ASNS has been found to be highly expressed in different subtypes of tumors [6] and its overexpression promotes cell proliferation, chemoresistance and metastasis [27]. ASNS dysfunction has been clinically associated with ASNS deficiency (ASD) and childhood acute lymphoblastic leukemia. In solid tumors, elevated ASNS expression is correlated with poor overall survival of lung cancer (www.proteinatlas.org). Moreover, ASNS over expression can prevent cell apoptosis and cycle arrest owing to ATF4 deficiency or nutrition stress [9].

The 3D structure of the AS-B enzyme from *E*. *coli* has been determined and refined to 2.0 Å resolution [10]. The side-chain functional groups of Arg49, Asn74, Glu76, and Asp98 form hydrogen bonds with the Gln ligand. These hydrogen bonds range in length from 2.6 to 3.1 Å. Unlike the active site in the glutaminase portion (the *N*-terminal region of asparagine synthetase), the *C-*terminal active site is less well-defined in the present complex. The synthetase domain of ASNS is responsible for the binding of Mg^2+^, ATP and Asp, that leads to formation of the beta-aspartyl intermediate. Val272, Leu232, Gly347, Ser346, Asp238, Asp351, Asp279 and Asp384 were observed as binding residues of *C*-terminal active site for ATP and Asp [7, 10].

In this work, *in silico* tools were used to define the different residues involved in the catalytic function of human ASNS. Initially ASNS FASTA sequences of both human and *E*. *coli* were downloaded and aligned by using the CLUSTAL OMEGA tool to identify the conserved regions (Fig 1). These results showed that the active site regions of both *N-*and *C*-terminal are almost conserved (more than 90%), making it an excellent candidate to define the human ASNS.

**Fig 13:**
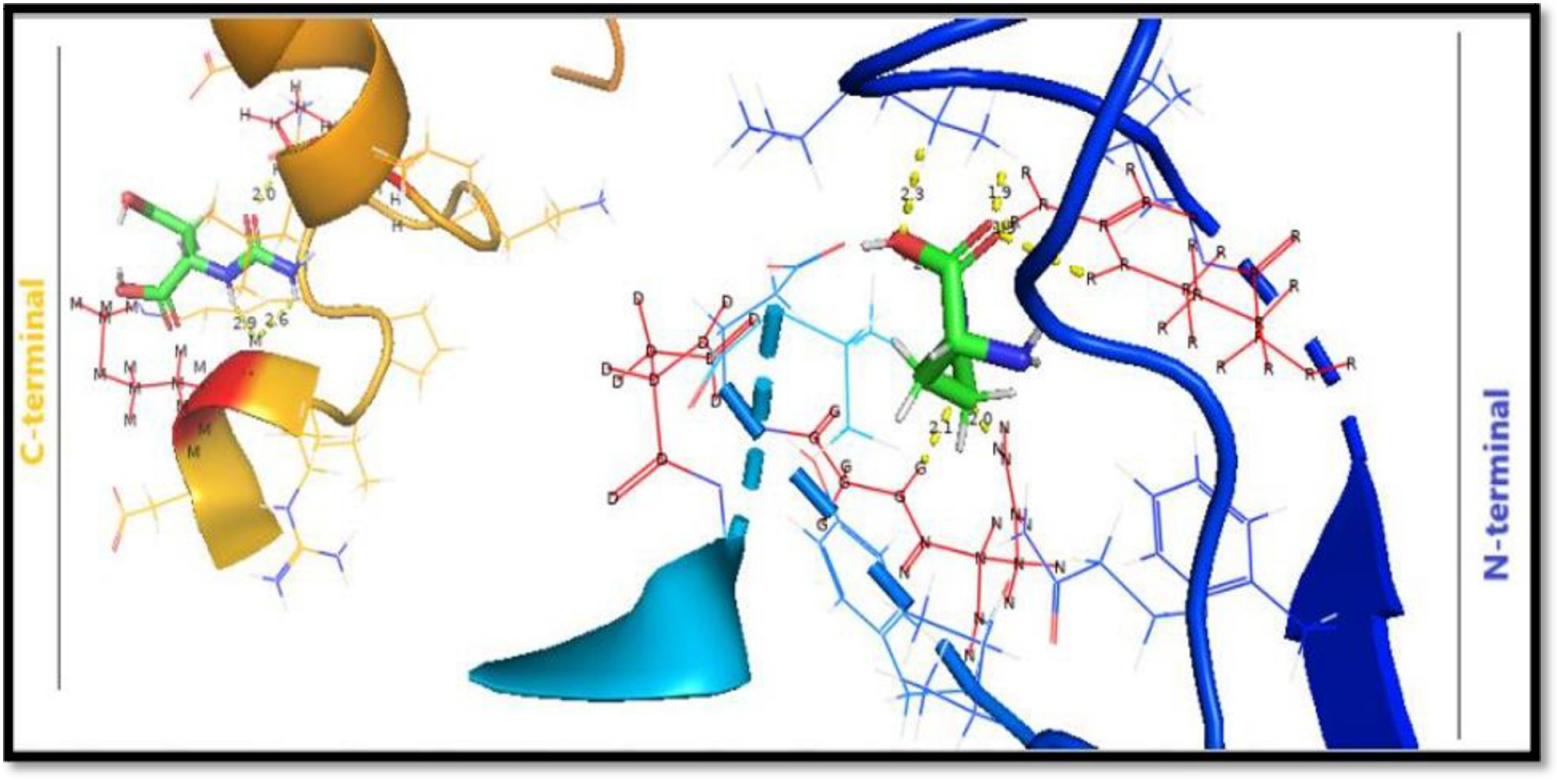
3D model development of human ASNS with *N-* and *C-*terminal active sites with their respective ligands.

The downloaded human ASNS structure from PDB, was prepared for docking by using the Pymol and Discovery studio software. The Autodock Vina tool was used to dock Asp, Gln, ATP and Beta Aspartyl AMP with human ASNS, one by one (S5 Table in S2 File). For each docking 10 models were produced. The best models were selected on the basis of their binding energy and RMSD values (Table 1). Different images were developed by using LIGPLOT, Pymol and Discovery studio, which shows how ASNS interacts with all the known substrates. These images show the interactions between the known substrates with specific amino acids. The 3D models of the proteins can be developed by using bioinformatics studies in the absences of experimental working to predict the structural relationship between enzyme and its ligand. These models are significant to depict structural annotations of an active site and different representations can be created to regions of interest in a protein [31]. In this work 3D model was created of human ASNS with its known substrates for both its active sites: Gln with glutaminase site and ATP with synthetase site. Both these ligands were used simultaneously to predict 3D models of the enzyme that depict the structural features and shows which amino acids are involved in binding with the respective ligands bind in the active sites of ASNS (Fig 13). The given figure showing cartoon representation of 3D ASNS enzymes having both ligand molecules, which is not yet published computationally or experimentally.

Docking between Gln and ASNS shows that Gln binds with Arg48(2.86 Å), Asn74(2.92 Å), Gly75(2.88 Å) and Glu414(2.99 Å) residues of *N-*terminal through Hydrogen bonds. Apart from these residues Val52, Val95, Tyr73, Cys1, Ala50 and Glu76 provide stability to the ligand through hydrophobic interactions (Fig 5).This is in agreement with Goto *et al*. [32], who has documented that when Gln binds to CTP synthetase in its active site the alpha carbonyl group of Gln binds via hydrogen with the amino groups of Arg and Gly, suggesting that Gln plays an important role in the intermolecular association with ASNS leading to release of ammonia from Gln.

Docking of ATP with ASNS shows binding of ATP with residues situated in the *C-*terminal domain of ASNS (Fig 6). These include Ser257(2.83 Å), Asp261(2.86 Å) Ser262(2.83 Å), Glu364(2.89 Å), Gly365(3.19 Å), Asp367(2.70 Å), Tyr373(2.91 Å), Asp400(2.97 Å), Arg403(2.92 Å), through hydrogen bonding and at Gly363, Gly343, Glu368, Phe314, Trp480, Ala340 and Met344 through hydrophobic interactions. ATP binding to ASNS releases PPi and AMP, which further reacts with Asp yielding β-aspartyl-AMP (Fig 8).

ASNS belongs to the family of ‘N-type’ATP pyrophosphatases, which also includes NAD synthetase, GMP synthetase and arginine-succinate synthetase. These enzymes play an important role in the hydrolysis of ATP, and their common reaction mechanism include nucleophile attack by either an ammonia generated from Gln (such as NAD-, GMP- or Asn synthetase) or in case of arginine-succinate synthetases the amino group of a reactant. Their active site shares structural similarity, which includes conserved residues such as Ser, Gly and Asp, when using ATP and Asp as substrates [33]. Mutation of Ser and Gly has been shown to interrupt the binding and disrupt the overall configuration of the active site [33], suggesting that these conserved residues are crucial for the hydrolysis of ATP. Furthermore, the three clusters of negatively charged amino acids Glu-364, Asp-367 and Glu-368 have been proposed to define the binding selectivity of an ASNS inhibitor [11], suggesting that these residues are crucial in binding of molecules such as ATP.

When Asp binds the ASNS enzyme, it becomes activated in the presence of ATP, which leads to the formation of β-aspartyl-AMP. It is further converted to Asn in the presence of ammonia. Docking analysis between β-aspartyl-AMP and ASNS revealed that it binds via hydrogen bonds with Ser257(2.89 Å) and Gly363(2.94 Å). The amino acids Ser262, Ser362, Leu256, Met344, Ile287, Ile347 and Leu255 support this binding via hydrophobic interaction (Fig 8). Docking of Asp with ASNS shows that Asp binds with Glu219 (2.80Å) and His445 (2.84Å) via hydrogen bonds and His212, Asp216, Lys439, Met435, Glu434, Glu449, Ile227 and Leu446 stablized this bonding via hydrophobic interactions (Fig 7).

Based on this information, there is an urgent need to identify how all these substrates are bound to the active site of the enzyme ASNS. It would also be helpful to understand the complete mechanism of how Asp and Gln convert to Asn and Glu. This work may stimulate further development of inhibitors of ASNS to downregulate the synthesis of Asn, an important trigger of breast cancer metastasis.

MD simulation studies are mostly performed to determine the ligand-protein binding in physiological environment and to monitor their stability during a simulation timeline. In this study simulation studies were performed for docked complexes of protein with its natural substrates. The simulation results depict that all complexes obtained from docking results shows good stability, lower RMSD and RMSF value predict the reliability of the docking results. The *C*-terminal binding pocket residues seems to more stable and ATP docked complex show more contacts during simulation (24 contacts), which suggest that the *C*-terminal binding residues are more prominent and can be potential target points for inhibitor development [34].

ASNS inhibition can suppress breast cancer metastasis, as the ASNS enzyme produces Asn, which is directly involved in activation of various transcriptional factor such as SNAIL, TWIST WLAN. These transcription factors are mainly involved in breast cancer proliferation. For developing new inhibitors against an enzyme there is an urgent need to understand how an enzyme interacts with its substrates, its structure and function. In this study number of binding residues, which are involved in the interaction of ASNS with its own substrates were identified by computational docking tools. To validate these binding sites different known inhibitors (sulfoximine adenylate, phosmidosine, mupirocin and 8N3ATP) were retrieved from the BRENDA database for ASNS enzyme (E.6.3.4.5) [35, 36]. These inhibitors were also used to bind with ASNS by utilizing Autodock Vina.

The sulfoximine compounds are profoundly known for their anti-cancer activity. It has been reported that adenylated sulfoximine is ASNS inhibitors and adenylated sulfoximine 7b has been used to define the enzymes inhibition activity in MOT4 cell lines [37]. Sulfoximine adenylate was used to dock with the *C*-terminal region of human ASNS. The result showed that this compound binds through hydrogen bonds with Asp261, Gly363 and Ser362 found in the active site pocket of *C*-terminal region. The minimum binding energy of selected model was -8.3kcal/mol, and RMSD values from lower bound and upper bound were 0.000 (S5 Table in S2 File and S5 Fig in S1 File).

Phosmidosine and its derivatives are reported for their anticancer activity in tumor cells and inhbiting activity, which has been evaluated by MTT assay in various tumor cell lines [38]. Phosmidosine also inhibit prolyltRNA in breast cancer [39]. Docking of phosmidosine with ASNS shows that it binds with Asp261, Ser262, Ser362, Gly363 and Gly343 via hydrogen bonding found in *C*-terminal active site pocket of the enzyme. The minimum binding energy of model was found to be -8.7kcal/mol, and RMSD values from lower bound and upper bound were 0.000 (S5 Table in S2 File and S6 Fig in S1 File).

Mupirocin has anti cancerous activity against the MCF-7 breast cancer cell line [40]. Like other inhibitors mupirocin was also docked with human ASNS, which showed that it binds with enzyme via hydrogen bonds (Asp261, Gly363, Glu443, Ser292, Asn478 and Trp480). The minimum binding energy of model was -8.8 kcal/mol, and RMSD values from lower bound and upper bound were 0.000 (S5 Table in S2 File and S7 Fig in S1 File).

8-N3ATP (8-azidoadenosine 5′-triphosphate) is an ATP analog compound, which inhibits the ammonia dependent synthesis of Asn. The ASNS enzyme was chemically modified by using 8-N3ATP at the ATP binding pocket, leading to inhibition of its catalytic activity of ASNS [41]. 8-N3ATP retrieved from the pubchem database was used to dock with ASNS, which revealed that 8-N3ATP binds at *C*-terminal ATP binding pocket via hydrogen bonds (Asp261, Gly363, Glu368, Asp367, Glu364 and Ser366). The minimum binding energy of model was -8.8kcal/mol, and RMSD values from lower bound and upper bound were 0.000 (S5 Table in S2 File and S8 Fig in S1 File).

All the above-mentioned inhibitors were also used to dock with *N*-terminal active site of ASNS. The inhibitors did not bind in the catalytic domain and weak binding energy was found. The binding residues identified in this study suggest how the enzyme interacts with its natural substrates. Asp261, Gly363, Asp367 are considered as prominent bind site residues and have more potential for interaction with other compounds. These three amino acids directly interact via hydrogen bond to its natural substrates active site pocket of *C*-terminal. An experimental study was conducted on human ASNS in which X-ray determined structure of enzyme was analyzed by chemo-protemics profiling and they identified a group of amino acids chains which are negatively charged. They reported that these are prominent amino acids and can be used to define the binding ability of the inhibitors. This cluster is consisting on Glu and Asp and both are acidic and negatively charged amino acids [11].

A conclusive finding of this study shows that these residues can be used for developing new inhibitors to minimize the synthetase activity, Asn production of the enzymes without affecting its glutaminase activity. In future studies all the identified binding site residues especially Asp261, Gly363, Glu364 and Asp367 can be used as therapeutic targets for developments of new drugs or inhibitors to inhibit ASNS. When compared with previous approaches, where the ASNS enzyme of *E*. *coli* was studied and explained with its natural substrates, and the human ASNS 3D model was determined using the compound DON [11]. In this work the human ASNS 3D structure was used for further analysis with and prepared as a target for docking and simulation studies to clearly define the binding site residues of both active sites. The studied enzyme involves 4 natural substrates during the conversion reaction, as define residual wise interactions of all the natural substrate of the enzyme, which has not been determined before. Clinical relevance of ASNS has become more important in breast cancer research, because the production of non-essential Asn act as a fuel and governs metastasis by helping tumor cell migration and proliferation. To understand the enzyme binding patterns, interaction regions and inhibitory sites are clinically important in cancer treatment. Prediction of potential amino acids for enzyme inhibition is crucial to meet the new trends in pharmaceuticals for drug development.

## Supporting information

S1 File 3D models of the Gln, ATP, Asp and β-Aspartyl AMP docking with ASNS. Ligplot graphs showing hydrogen bonding and hydrophobic interaction between Sulfoxime, Phosmidosine, Mupirocin and 8N3ATP with ASNS protein.
(DOCX)

S2 File Log table of docking complex: Gln, ATP, Asp and β-Aspartyl AMP with ASNS. Binding residues of docking complexes between all substrates and ASNS and binding residues of docking complexes between all ligands and ASNS.
(DOCX)

S3 File (PDF)
