# Supplementary material for: In silico approaches to study the human asparagine synthetase: An insight of the interaction between the enzyme active sites and its substrates
Source: PLoS One. 2024 Aug 2;19(8):e0307448. doi: 10.1371/journal.pone.0307448 (PMC11296641; doi:10.1371/journal.pone.0307448)
Supplement: S1 File — Ligplot graphs showing hydrogen bonding and hydrophobic interaction between Sulfoxime, Phosmidosine, Mupirocin and 8N3ATP with ASNS protein. (DOCX) [file pone.0307448.s001.docx]

**Supplementary Figures**

**ASNS+Gln**

**
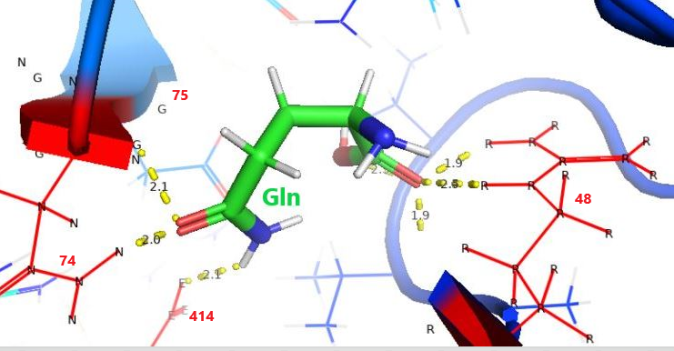
**

**(A)**

**
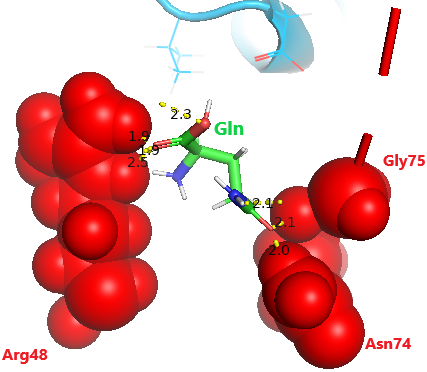
**

**(B)**

**
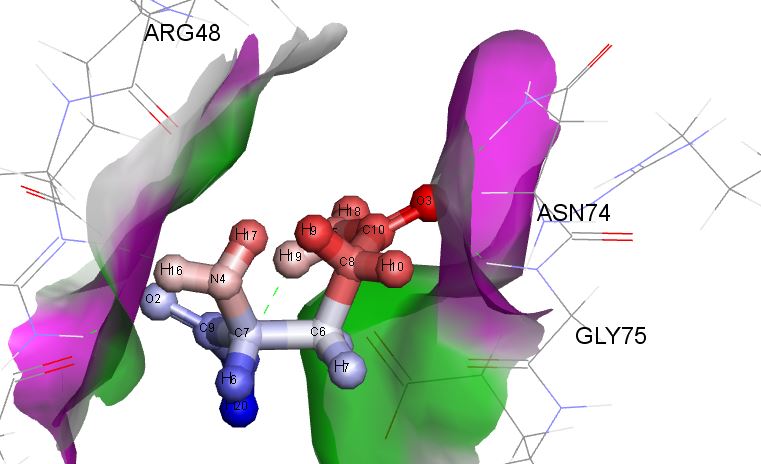
**

**(C)
Fig S1. 3D Model development of Gln docking with ASNS.**

**(**A) Showing cartoon structure (B) showing ball and stick model (C) Showing binding pocked covered Gln

**ASNS+ATP**


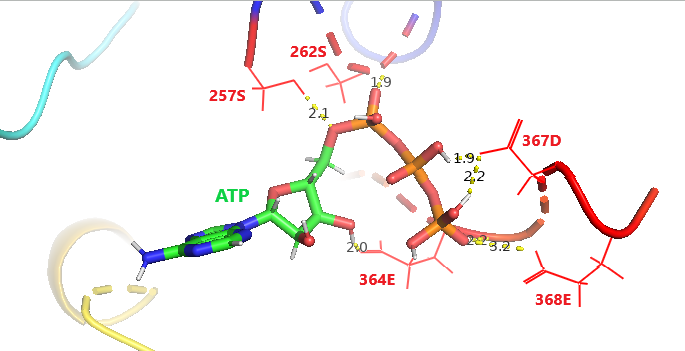


**(A)**


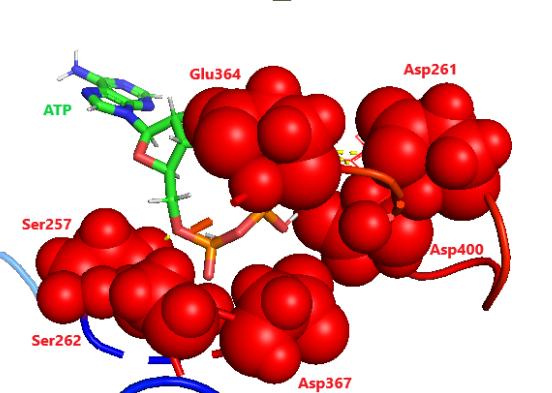


**(B)**


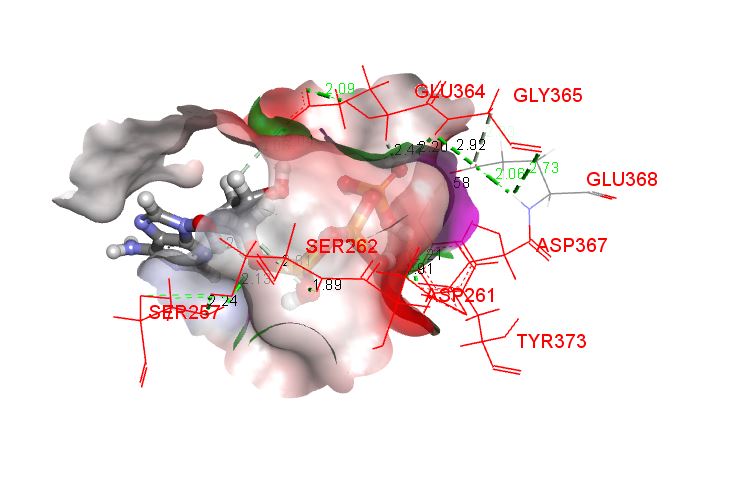


**(C)**

**Fig S2. 3D Model development of ATP docking with ASNS.**

**(**A) Showing cartoon structure (B) showing ball and stick model (C) Showing binding pocked covered ATP

**ASNS+Asp**

**
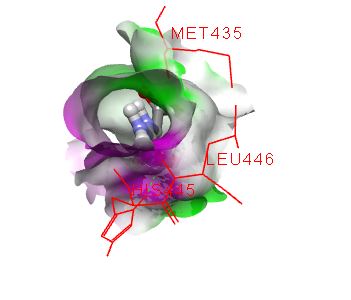
**

**(A)**

**
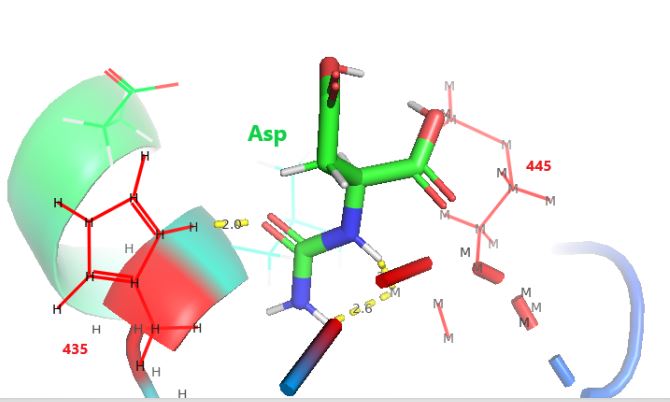
**

**(B)**

**
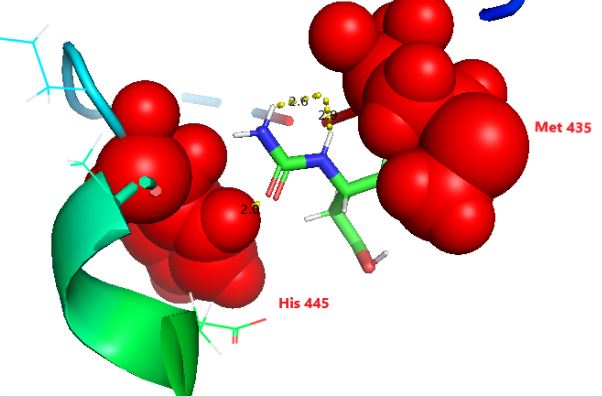
**

**(C)**

**Fig S3. 3D Model development of Asp docking with ASNS.**

**(**A) Showing cartoon structure (B) showing ball and stick model (C) Showing binding pocked covered Asp

**ASNS+Beta Asp AMP**

**
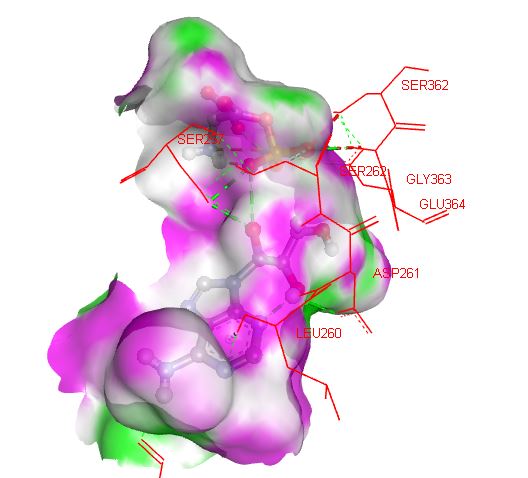
**

**(A)**

**
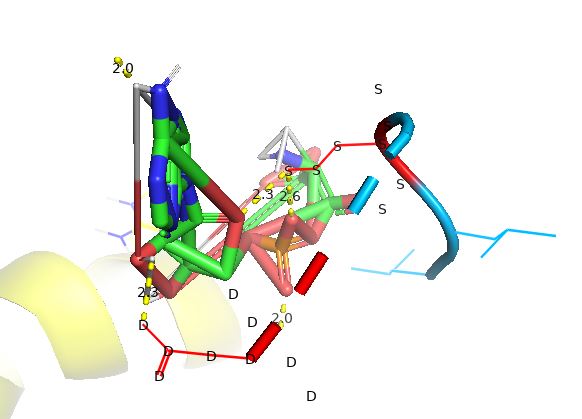
**

**(B)**

**
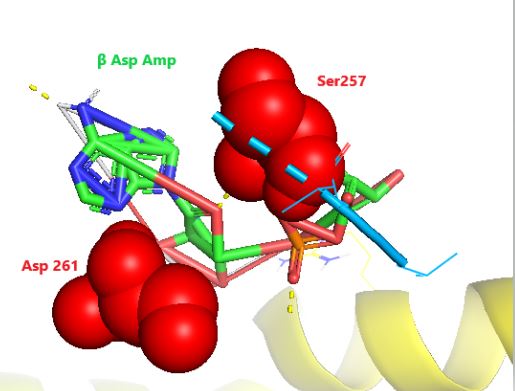
**

**(C)**

**Fig S4. 3D Model development of β-Aspartyl AMP** **docking with ASNS.**

**(**A) Showing cartoon structure (B) showing ball and stick model (C) Showing binding pocked covered beta Asp AMP

**
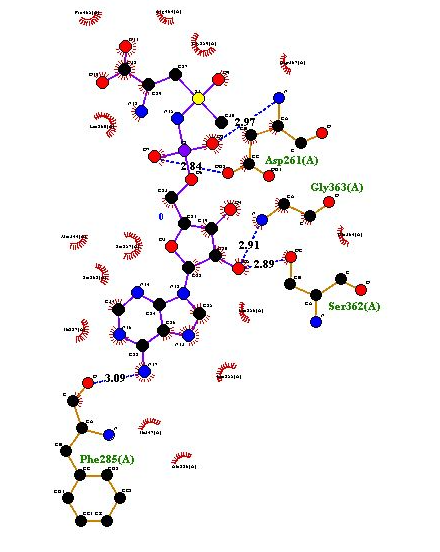
**

**Fig S5. Ligplot showing hydrogen bonding and hydrophobic interaction between Sulfoxime adenylate and ASNS.**


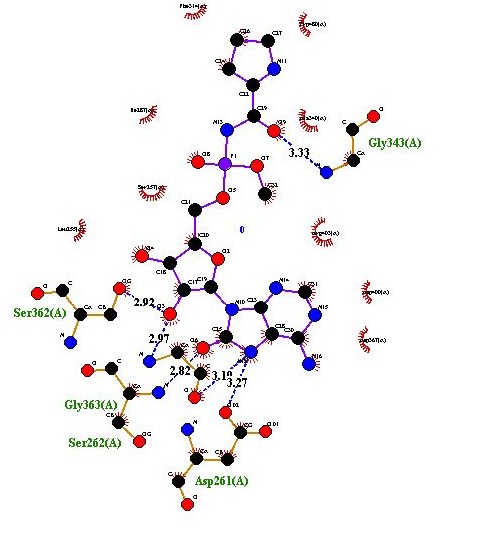


**Fig S6. Ligplot showing hydrogen bonding and hydrophobic interaction between Phosmidosine and ASNS protein.**

**
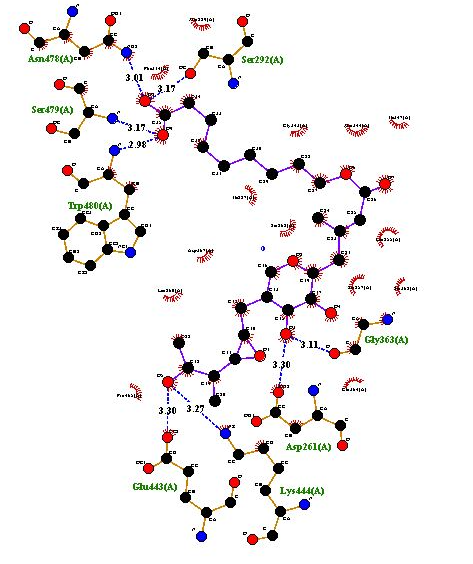
**

**Fig S7. Ligplot showing hydrogen bonding and hydrophobic interaction between Mupirocin and ASNS**

**
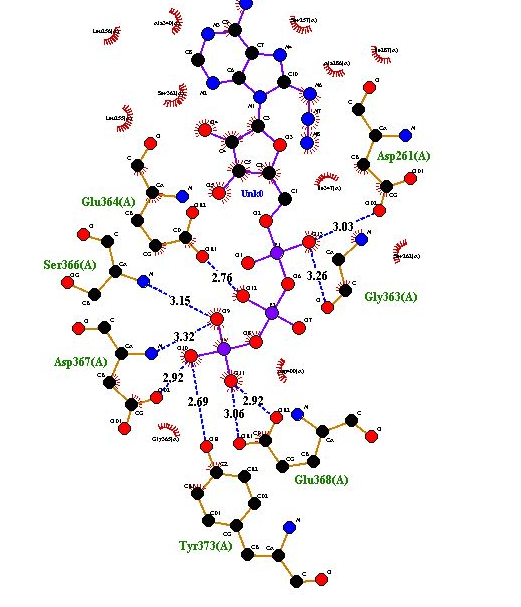
**

**Fig S8. Ligplot showing hydrogen bonding and hydrophobic interaction between the 8N3ATP and ASNS .**
