# Supplementary material for: In silico approaches to study the human asparagine synthetase: An insight of the interaction between the enzyme active sites and its substrates
Source: PLoS One. 2024 Aug 2;19(8):e0307448. doi: 10.1371/journal.pone.0307448 (PMC11296641; doi:10.1371/journal.pone.0307448)
Supplement: S2 File — Binding residues of docking complexes between all substrates and ASNS and binding residues of docking complexes between all ligands and ASNS. (DOCX) [file pone.0307448.s002.docx]

**Supplementary Tables**

**Table S1: Log table of docking complex: Gln and ASNS**

**
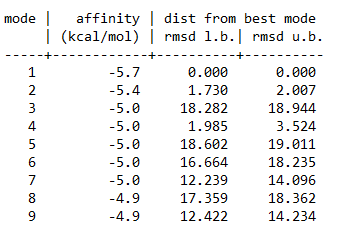
**

**Table S2: Log table of docking complex: ATP and ASNS**

**
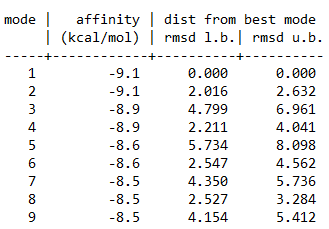
**

**Table S3: Log table of docking complex: Asp and ASNS**

**
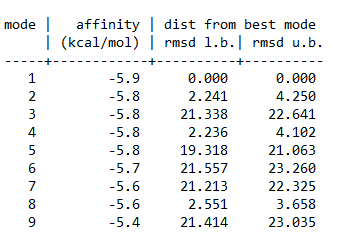
**

**Table S4: Log table of docking complex: β-Aspartyl AMP and ASNS**

**
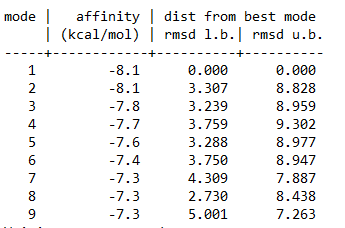
**

**Table S5: All the binding residues of docking complexes between all substrates and ASNS**

| **Docking Complex** | **Model No** | **Binding Energy**  (kcal/mol) | **RMSD** | **Binding Residues** | |
| --- | --- | --- | --- | --- | --- |
|  |  |  |  | Residues with Hydrogen bond & bond length | Residues with hydrophobic interaction |
| ASNS with Gln | 01 | -5.7 | 0.000 | Arg48 (2.86 Å)  Asn74(2.92 Å)  Gly75 (2.88 Å)  Glu414(2.99 Å) | Val95,  Tyr73  Leu49  Val52 |
| ASNS with ATP | 01 | -9.1 | 0.000 | Glu364(2.89Å)  Ser257(2.83Å)  Ser262(2.83Å)  Gly365(3.19Å)  Asp400(2.97Å)  Arg403(2.92Å)  Asp367(2.70Å)  Asp261(2.86Å) | Gly259  Gly363  Gly343  Met344  Glu368  Val342 |
| ASNS with Asp | 01 | -5.9 | 0.000 | Met435(3.00Å)  His445(3.03Å) | Ile437  Pro437  Asp216  Leu446 |
| ASNS with AMP | 01 | -8.5 | 0.000 | Gly363(2.84Å)  Gly365(3.14Å)  Glu368(2.98Å) Asp400(3.31Å)  Asp367(2.88Å) | Glu364  Asp261  Ile347  Ile287  Gly343 |
| ASNS with β-Aspartyl AMP | 01 | -8.1 | 0.000 | Ser257(2.89Å)  Gly363(2.94Å) | Ser  Leu  Ile |
|  | 02 | -8.1 | 3.307 | Asp261(3.0 Å)  Leu260(2.98Å)  Lys444(2.95Å)  Arg448(3.29Å)  Pro465(3.24 Å) | Gly  Glu |

**Table S6: All the binding residues of docking complexes between all ligands and ASNS**

| **Ligand** | **Target region of ASNS** | **Model** | **RMSD** | **Binding Energy**(kcal/mol) | **Binding Residues** |
| --- | --- | --- | --- | --- | --- |
| **Sulfoximine Adenylate** | *C-*terminal | 01 | 0.000 | -8.3 | Asp261  Gly363  Ser362 |
| **Phosmidosine** | *C-*terminal | 01 | 0.000 | -8.7 | Asp261  Ser262  S362  Gly363  Gly343 |
| **Mupirocin** | *C-*terminal | 01 | 0.000 | -8.8 | Asp261  Gly363  Glu443  Ser292  Asn478  Trp480 |
| **8-N3ATP** | *C-*terminal | 01 | 0.000 | -8.8 | Asp261  Gly363  Glu368  Asp367  Glu364  Ser366 |
