# Supplementary figures and images for: In silico approaches to study the human asparagine synthetase: An insight of the interaction between the enzyme active sites and its substrates
Source: PLoS One. 2024 Aug 2;19(8):e0307448. doi: 10.1371/journal.pone.0307448 (PMC11296641; doi:10.1371/journal.pone.0307448)

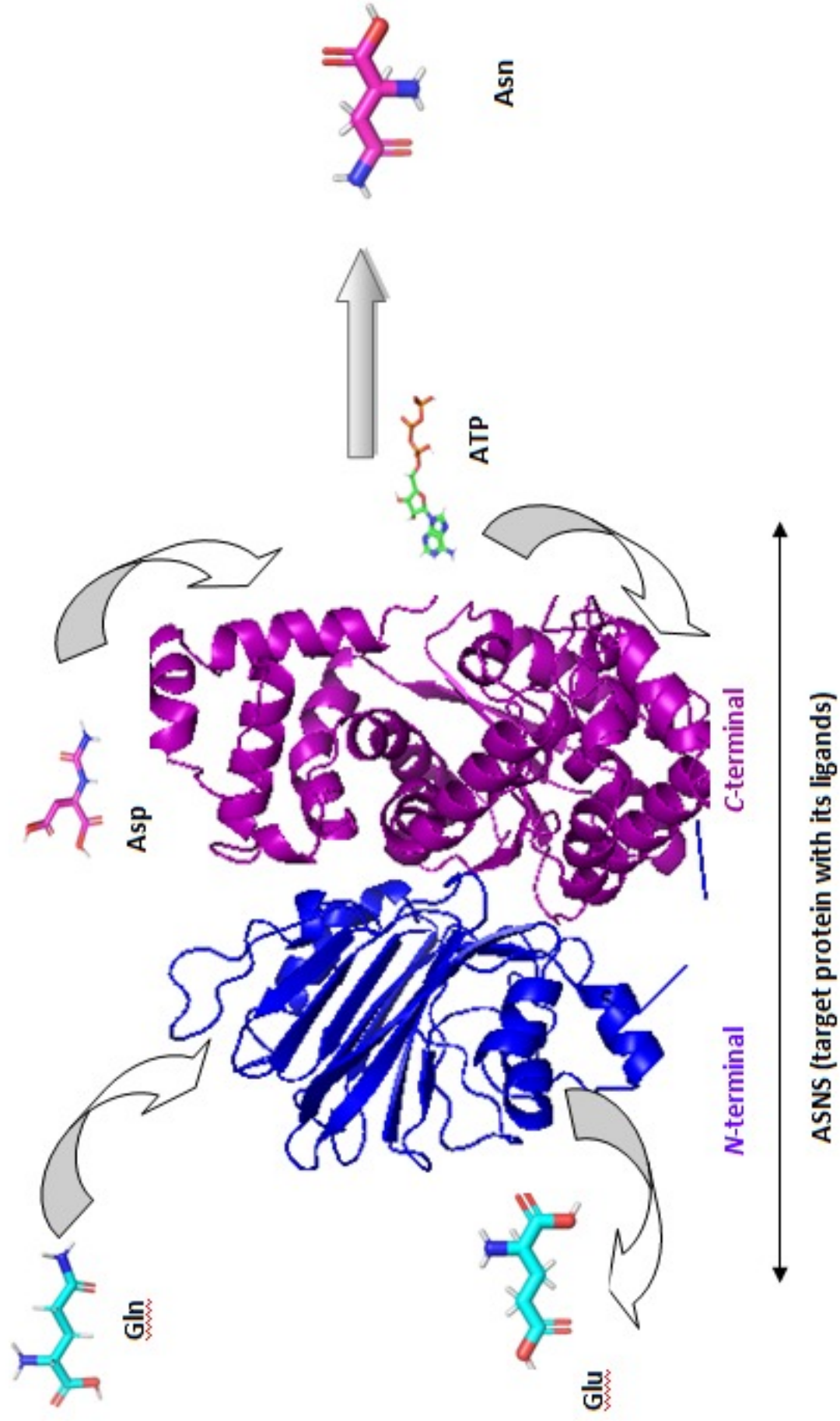

Supplement: S3 File — (PDF) [file pone.0307448.s003.pdf]
